# The invasive MED/Q *Bemisia tabaci* genome: a tale of gene loss and gene gain

**DOI:** 10.1186/s12864-018-4448-9

**Published:** 2018-01-22

**Authors:** Wen Xie, Xin Yang, Chunhai Chen, Zezhong Yang, Litao Guo, Dan Wang, Jinqun Huang, Hailin Zhang, Yanan Wen, Jinyang Zhao, Qingjun Wu, Shaoli Wang, Brad S. Coates, Xuguo Zhou, Youjun Zhang

**Affiliations:** 1 Department of Entomology, Institute of Vegetables and Flowers, Chinese Academy of Agricultural Science, No. 12 Zhongguancun Nandajie, Haidian District, Beijing, 100081 China; 2 BGI-Shenzhen, Shenzhen, 518083 China; 3 United States Department of Agriculture, Agricultural Research Service, Corn Insects & Crop Genetics Research Unit, Ames, IA 50011 USA; 4 Department of Entomology, University of Kentucky, S-225 Agricultural Science Center North, Lexington, KY 40546-0091 USA

**Keywords:** *Bemisia tabaci*, MED/Q, Invasive species, Genome, Gene gain and loss, Detoxification enzymes, Symbiosis

## Abstract

**Background:**

Sweetpotato whitefly, *Bemisia tabaci* MED/Q and MEAM1/B, are two economically important invasive species that cause considerable damages to agriculture crops through direct feeding and indirect vectoring of plant pathogens. Recently, a draft genome of *B. tabaci* MED/Q has been assembled. In this study, we focus on the genomic comparison between MED/Q and MEAM1/B, with a special interest in MED/Q’s genomic signatures that may contribute to the highly invasive nature of this emerging insect pest.

**Results:**

The genomes of both species share similarity in syntenic blocks, but have significant divergence in the gene coding sequence. Expansion of cytochrome P450 monooxygenases and UDP glycosyltransferases in MED/Q and MEAM1/B genome is functionally validated for mediating insecticide resistance in MED/Q using in vivo RNAi. The amino acid biosynthesis pathways in MED/Q genome are partitioned among the host and endosymbiont genomes in a manner distinct from other hemipterans. Evidence of horizontal gene transfer to the host genome may explain their obligate relationship. Putative loss-of-function in the immune deficiency-signaling pathway due to the gene loss is a shared ancestral trait among hemipteran insects.

**Conclusions:**

The expansion of detoxification genes families, such as P450s, may contribute to the development of insecticide resistance traits and a broad host range in MED/Q and MEAM1/B, and facilitate species’ invasions into intensively managed cropping systems. Numerical and compositional changes in multiple gene families (gene loss and gene gain) in the MED/Q genome sets a foundation for future hypothesis testing that will advance our understanding of adaptation, viral transmission, symbiosis, and plant-insect-pathogen tritrophic interactions.

**Electronic supplementary material:**

The online version of this article (10.1186/s12864-018-4448-9) contains supplementary material, which is available to authorized users.

## Background

The sweetpotato whitefly, *Bemisia tabaci,* consists of a group of cryptic sibling species [1] that contains some of the world’s most damaging agricultural pests, and are also considered among the world’s worst invasive species (Global Invasive Species Database: http://www.issg.org/database/welcome/) [2]. This impact on global agriculture is likely due to their broad host range (reported to feed on over 900 plant species) and transmit plant diseases (vectoring over 100 plant viruses) [3–6]. Within the cryptic species group, *Bemisia* Middle East-Asia Minor 1 (MEAM1, or ‘B’) and *Bemisia* Mediterranean (MED, or ‘Q’) are the two most extensively studied, and they have emerged as a comparative model for research into biological habitat invasion [1, 5–7], virus-vector interaction [4], symbiosis-host interaction and haplodiploid sex determination [8, 9]. Although both MEAM1/B and MED/Q are highly invasive, the two subspecies prefer different hosts, and also differ in their responses to virus-infected plants [10, 11] and capacity to vector plant viruses [7, 12]. The success of MEAM1/B as an invasive species may be related to its high ratio of diploid female to haploid male progeny, a competitive reproductive strategy that allows numerical displacement of native *Bemisia* species [5]. In contrast, MED/Q shows high levels of resistance to many classes of synthetic insecticides, providing a different strategy in survival within agricultural and other highly managed landscapes [7].

*Bemisia* and other phloem-feeding hemipterans rely heavily upon obligate bacterial endosymbionts in order to provide the essential amino acids and vitamins lacking in nutritionally incomplete plant sap [13–15]. All known *Bemisia* cryptic species have co-evolved with the intracellular primary endosymbiont, *Candidatus Portiera aleyrodidarum*. *Portiera* is transmitted maternally, and complements host’s unbalanced diets [16, 17]. Whiteflies also harbor secondary symbionts that are not strictly required for host survival and reproduction, but can exert a variety of effects on their whitefly hosts, including developmental time and fecundity [18, 19], susceptibility to insecticides [20], and modified vector competency [21–23]. *Bemisia*-bacteria symbiosis has evolved into an inter-dependent biosynthetic pathways for supplying required enzymatic components to metabolic pathways via complementation. The adaptive changes in host immune pathways and pathogen detection, and the mechanisms by which endosymbiotic bacteria evade these defenses, remains poorly understood. Moreover, variation in endosymbiont communities within *Bemisia* is associated with distinct host haplotypes, and these endosymbiont communities may influence the competency of *Bemisia* subspecies to vector different plant viruses [23].

Motivated by the invasive nature and economic impacts on agricultural crop production, draft genome assemblies have recently been completed for MEAM1/B [24] and MED/Q [25]. Genome sequences of MEAM1/B provide insights into evolutionary and adaptive mechanisms by which the whitefly has become such a formidable threat to global food security. Specifically, this was predicted based on the mining of gene families putatively involved in insecticide resistance, xenobiotic detoxification, virus transmission, and horizontally transferred genes [24]. Our previously published draft MED/Q genome contained assembly and annotation information [25], but within the current study we take a whole genome approach to comprehensively compare the divergence in gene sequences and contents between MED/Q and MEAM1/B. These analyses focus on characterizing the degree and nature of horizontal gene transfer resulting from co-evolution with endosymbionts, as well as the genomic changes which may contribute to the selective advantages of MED/Q and MEAM1/B within the context of an invasive agricultural pest species. Additionally, predicted adaptations of detoxification observed via lineage-specific expansions and contractions are functionally validated for potential roles in insecticide tolerance in MED/Q. This research is important for understanding the possible role of functional diversification of recent gene family expansions may have on adaptive capacity among crop insect pests, as well as evolutionary processes that increase relative fitness of invasive insect populations.

## Results

### Genome comparison between the two invasive *B. tabaci* cryptic species

Results of comparison between genomes predicted that 16,523 (79%) of the genes in the MED/Q genome share similarity with 10,421 (66%) of genes in MEAM1/B (Additional files 1 and 2). In addition, mapping of reads from a MED/Q 500 bp insert size library (PE100) to MED/Q and MEAM1/B genomes, using SOAPaligner/soap2 demonstrated a difference in proportion of reads that aligned (Additional file 3: Table S1). Specifically, the proportion of MED/Q reads that mapped to MED/Q (79.2%; 69.55% for paired alignment ratio, plus 9.65% for singled alignment ratio), was greater compared to the proportion that mapped to MEAM1/B (56%; 26.63%, plus 30.04%) sequencing reads from MED/Q mapping to. To study the syntenic blocks between these two genomes, an alignment of MED/Q and MEAM1/B draft genome assemblies was performed using *LastZ* [26]. An estimated 77.94% of the MED/Q and 83.22% of the MEAM1/B genomes were aligned into syntenic blocks (Additional file 4: Table S2). This showed that on average 5.26% of nucleotide sequence across the conserved regions of the MED/Q genome to which reads were mapped comprised substitutions when compared to MEAM1/B (Additional file 4: Table S2). A total span of 2.91% of these aligned read lengths of both the MED/Q and MEAM1/B genome were indels. In summary, the per-nucleotide sequence divergence between MED/Q and MEAM1/B was estimated at 8.17% in MED/Q and 8.19% in MEAM1/B (Additional file 4: Table S2).

A total of 10 scaffolds in MEAM1/B with the greatest lengths were picked in order to provide visual exemplars of the predicted syntenic blocks (Additional file 5: Figure S1), and indicated that additional intervening non-homologous sequence residing within the MED/Q between syntenic regions. These analyses also demonstrated the comparatively fragmented nature of the MED/Q assembly and the capacity to estimate order and orientation of MED/Q scaffolds when using the MEAM1/B assembly as a reference. Comparison of the percent divergence among the coding sequences of orthologous 7794 genes in MED/Q and 7202 in MEAM1/B (Additional file 6: Table S3). Among these, 4052 pairs were putatively single copy (1:1) orthologs. Evidence of possible purifying or positive selection was estimated rates of nonsynonymous (Ka) and synonymous (Ks) substitutions between MED/Q and MEAM1/B in 4052 pairs one-to-one ortholog pairs. Among these, a Ka and a Ks rate could be calculated for 2985 ortholog pairs (Additional file 7), where the resulting mean values of Ka, Ks, and Ka/Ks were 0.031, 0.237 and 0.236, respectively. A total of 59 orthologous pairs had a Ka/Ks ratio > 1, and were interpreted as possible signs of positive selections within a given CDS, (Additional file 8), and functional annotations demonstrated the significant enrichment of gene products involved in DNA replication, proteasome and hematopoietic cell lineage processes (Additional files 9 and 10).

### Genome-based phylogeny and gene phylogenies

The evolutionary trajectory of MED/Q was investigated using a genome-based phylogenetic analysis pipeline carried out using 222 single-copy orthologous genes across the genomes of 14 insect species that used *Daphnia pulex* as an outgroup (Additional file 11: Figure S2). This resulted in the estimated mean divergence time of 95.6 million years between MED/Q and MEAM1/B across all 222 aligned orthologs (range 42.9 to 168.5 million years). Expansion of the gene sets under consideration predicted that 1925 orthologous gene families are specific to MEAM1/B in MED/Q (Additional file 12), in which, 887 are specific to all other 15 species (Additional file 13). These orthologous gene families showed putative annotations in membrane (GO: 0016020), transport (GO: 0006810), oxidoreductase activity (GO: 0016491) and receptor activities (GO: 0004872) among others (Additional files 14 and 15: Tables S4 and S5). Evidence for adaptive evolution was detected using the positively selected genes in the MED/Q and MEAM1/B lineage when compared to other phloem feeding insects (*Acyrthosiphon pisum*, *Nilaparvata lugens* and *Diaphorina citri*). From these analyses, 142 candidate positively selected genes were identified in MED/Q, which showed putative functional annotations for protein phosphorylation (GO: 0006468), protein kinase (GO: 0004672) and intracellular signal transduction activities (GO: 0035556) (Additional file 16; Additional file 18: Table S6). Moreover, 70 candidates mostly involved in signal transduction (GO: 0007165), ion channel activity (GO: 0005216, GO: 0006811) and acetylcholine-activated cation-selective channel activity (GO: 0004889) were shared in MED/Q and MEAM1/B branch (Additional file 17; Additional file 18: Table S6).

### Expansion and contraction of gene families

**Fig. 1:**
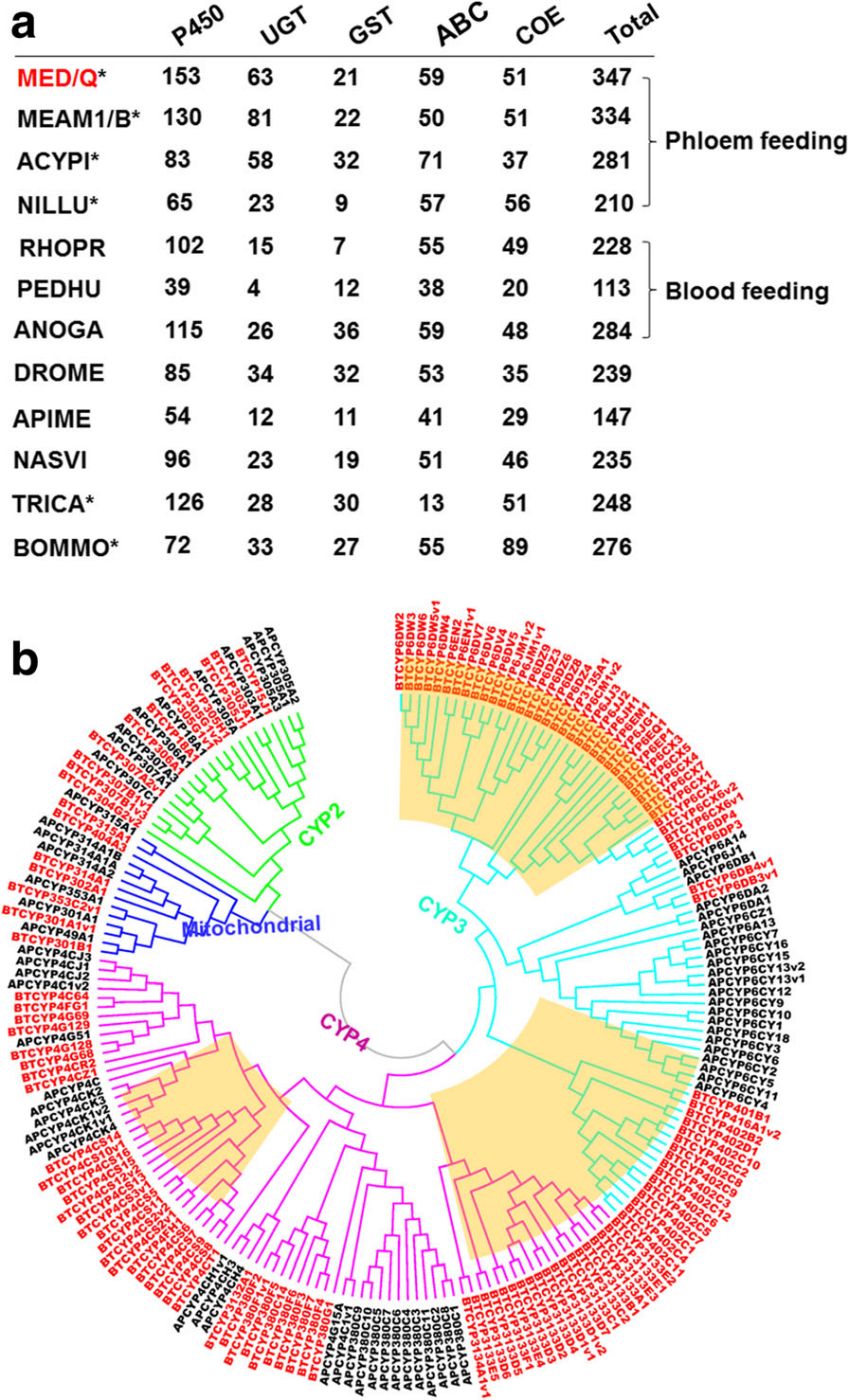
Expansion of gene families associated with metabolism and detoxification in MED/Q genome. **a** Number of detoxification-related genes in the genomes of 12 selected insects (*denotes herbivorous insects) annotated as UDP glycosyltransferases (UGT), glutathione S-transferase (GST), ATP-binding cassette (ABC) transporter, and, carboxyl/choline esterases (COE). **b** Neighbor-joining phylogeny of the cytochrome P450 monooxygenase genes between BEMTA (red) and ACYPI (black). Four insect CYP clades indicated as follows: CYP2 (green), CYP3 (blue green), CYP4 (pink), and the mitochondrial clade (blue). Predicted MED/Q-specific expansions within the CYP gene family are indicated (orange boxes). Q-type *Bemisia tabaci* (MED/Q), B-type *Bemisia tabaci* (MEAM1/B), *Acyrthosiphon pisum* (ACYPI), *Nilaparvata lugens* (NILLU), *Rhodnius prolixus* (RHOPR), *Pediculus humanus* (PEDHU), *Anopheles gambiae* (ANOGA), *Drosophila melanogaster* (DROME), *Apis mellifera* (APIME), *Nasonia vitripennis* (NASVI), *Tribolium castaneum* (TRICA) and *Bombyx mori* (BOMMO)

A total of 2943 gene families in MED/Q showed putative gene family member expansions when compared across the breath of species in the genomic phylogeny, of which the number of members within 20 of these gene families (20 of 2943; 0.7%) were significantly different (*p* ≤ 0.05). The largest copy number expansions occurring in gene families where members were assigned functional annotations as essential for membrane and transmembrane transport (Additional file 19: Table S7 and Additional file 20: Figure S3). Additionally, comparisons between MED/Q and MEAM1/B predicted that 32 of 722 gene families (32 of 755; 4.6%) show significant size expansions (*p* ≤ 0.05) (Additional file 20: Figure S3). Besides membrane and transmembrane transport function, these gene families under expansion in the MED/Q and MEAM1/B branch are associated with oxidation-reduction processes and monooxygenase activity (Additional file 21: Table S8). The number of gene families involved in metabolism and detoxification were similar between MED/Q and MEAM1/B (Fig. 1a). When expanding to scope of these comparisons, the number of MED/Q genes within the UDP glycosyltransferases (UGTs; *n* = 63), carboxyl/choline esterases (COE; *n* = 51), and ATP-binding cassettes transporters (ABC; *n* = 59) was not significantly different from those found in other phloem- or blood-feeding arthropods. In contrast, the cytochrome monooxygenase P450 detoxification gene family was significantly expanded in MED/Q and MEAM1/B (Fig. 1a). These expansions have produced 153 predicted MED/Q CYP genes, with the most expansions occurring in the CYP3 and CYP4 clades; this was greater compared to the number predicted in any other arthropod genome that was analyzed (Fig. 1b).

**Fig. 2:**
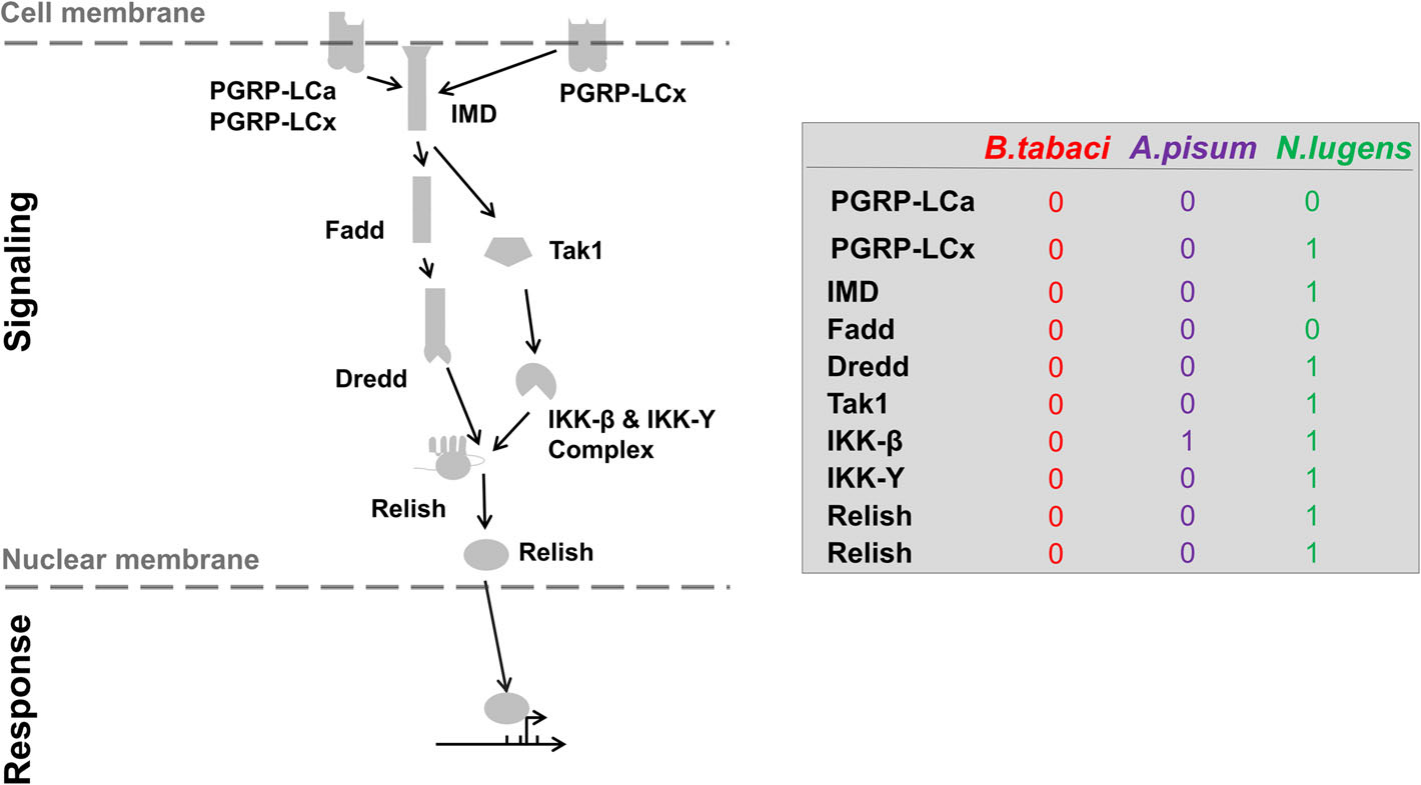
Predicted orthologues associated with immune deficiency (IMD) within hemipterans. Schematic diagram illustrates the IMD signaling and the corresponding responses. The table shows the number of genes encoding each insect genome

Putative reductions in the number of members in 1936 gene families were predicted in MED/Q, in which 12 gene families (12 of 1936; 0.6%) were significantly changed (*p* ≤ 0.05). These 12 gene families were primarily annotated as being involved in RNA binding, RNA-directed DNA polymerase activity and RNA-dependent DNA replication (e.g., difference in transposon component of the genomes), and serine-type endopeptidase activity (Additional file 22: Table S9). Within the MED/Q and MEAM1/B branch, 10 gene families were significantly contracted (*p* ≤ 0.05), and were mainly annotated as having involvement in functions similar to that predicted in MED/Q (Additional file 23: Table S10). Further inspection of gene annotation information showed that genes in the immune deficiency (IMD) pathway were absent from the MED/Q genome when compared to the genome of the hemipteran insect *N. lugens* (Fig. 2; Additional file 24: Table S11).

### In silico metagenomic analysis of MED/Q endosymbiosis

The bacterial metagenome of insects may contribute an important role in the overall fitness and viability of insects, such that many hemipterans have evolved specialized structures that house endosymbiotic bacteria called bacteriocytes. Although genomes are available for both the primary MED/Q endosymbiont, Ca. *Portiera* aleyrodidarum (CP003867, CP003835 and CP007563) and secondary endosymbiont, *Hamiltonella* (AJLH00000000, AJLH02000000) [13, 17], the lack of a corresponding whitefly genome sequence has precluded prior investigations into interactions with respect to metabolic and gene pathway partitioning. We used a metagenomics approach to re-assemble the complete genomes of *Portiera* (0.35 Mb) and *Hamiltonella* (1.8 Mb) from filtered Illumina reads generated from shotgun sequencing libraries.

**Fig. 3:**
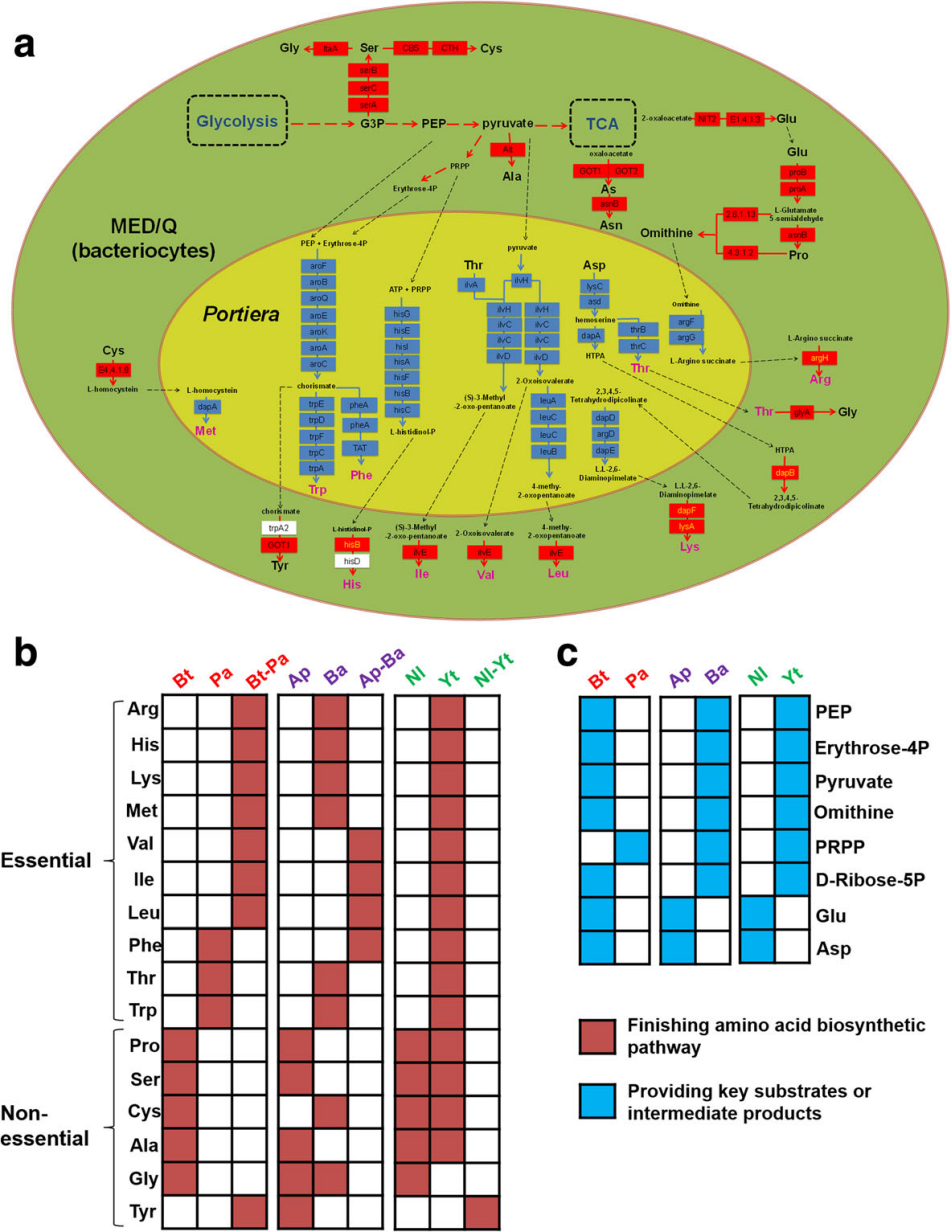
Comparative analysis of amino-acid biosynthesis and provisioning mechanisms in *B. tabaci, A. pisum* and *N. lugens*. **a** Unique amino acid biosynthetic and supply mechanisms putatively related to the adaptation of MED/Q. Green and yellow areas denote bacteriocytes and endosymbiont cells (with respect to the filtered and annotated *Portiera* genome of MED/Q, PRJNA299729), respectively. Essential amino acids are represented in pink and non-essential amino acids in black; *Portiera* genes are in blue boxes. The Enzyme Commission numbers (EC) or enzyme names used correspond to those in the Kyoto Encyclopedia of Genes and Genomes (KEGG). MED/Q genes are indicated in red boxes. Black dotted lines represent transport processes between MED/Q and *Portiera*, and red dotted lines represent processes associated with MED/Q that occur within *Portiera* bacteriocytes. Candidate horizontally-transferred genes (HTGs) are highlighted in yellow text; white boxes with black text represent unidentified genes. **b** Comparisons of amino acid biosynthesis in the host-symbiont bacterial systems of *B. tabaci*-*Portiera*, *A. pisum*-*Buchnera*, and *N. lugens*-yeast-like organism. Abbreviations: *Bt-Bemisia tabaci*, *Ap-Acyrthosiphon pisum, Nl-Nilaparvata lugens, Pa-Portiera, Ba-Buchnera, Yt-yeast-like*. The notation *Bt* (*Ap, Nl, Pa, Ba,* or *Yt*) means that MED/Q alone can complete the amino acid biosynthesis. The notation *Bt-Pa* (*Ap-Ba* or *Nl-Yt*) means that both MED/Q and at least two of its endosymbionts are required to complete the amino acid biosynthetic pathway. **c** Comparison of key substrates or intermediate products of the host-endosymbiont systems of *B. tabaci*-*Portiera, A. pisum-Buchnera* and *N. lugens*-yeast-like symbiont, illustrating that phosphoenolpyruvic acid (PEP), erythrose-4P, pyruvate, ornithine, and the precursor of histidine synthesis (PRPP) are important for amino acid synthesis. Pyruvate and PEP are produced by glycolysis and erythrose-4P by the pentose phosphate pathway. D-ribose-5P is the substrate for PRPP synthesis, and D-ribose-5P was converted based on D-glyceraldehyde 3-phosphate, also a product of glycolysis. Black arrows with dotted lines represent transport processes between MED/Q and *Portiera*

**Fig. 4:**
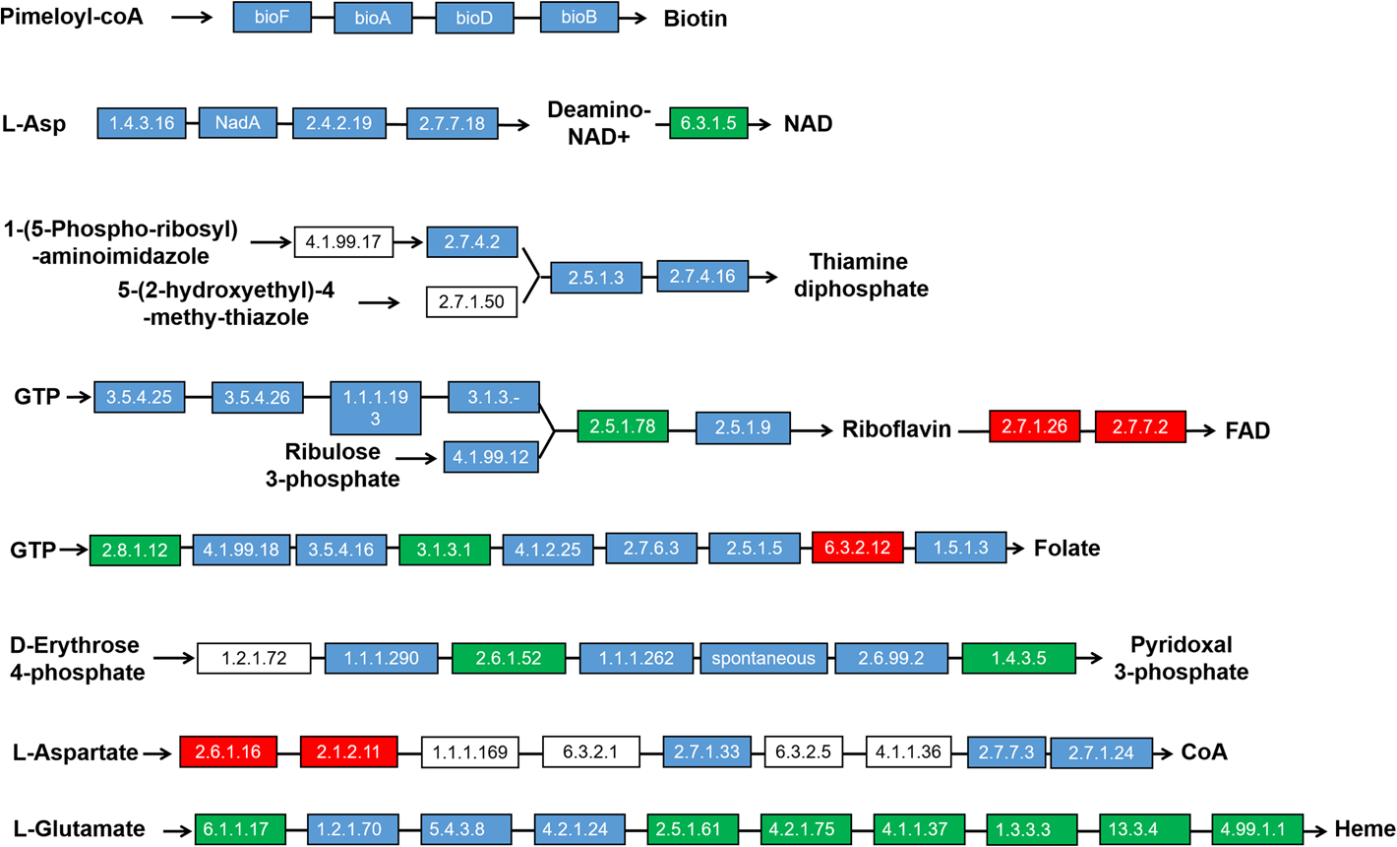
Pathways encoded by *Candidatus hamiltonella* for vitamin biosynthesis. Independence and complementarity in the vitamin synthesis pathways of MED/Q (red box) and *Hamiltonella* (blue box). *Hamiltonella* genes are highlighted in blue boxes with names corresponding to its genome (PRJNA299727), green boxes denote candidate genes encoded by both MED/Q and *Hamiltonella*, while white boxes indicate genes that do not have a match in either genome

An approach that used a comparison of MED/Q and endosymbiont gene models with functional annotations suggesting their involvement in amino acid biosynthesis to reconstruct the corresponding enzymatic pathways. The results of these comparative analyses revealed an interdependent relationship between MED/Q and *Portiera* biosynthetic pathways. Specifically, there were 47 MED/Q- and 45 *Portiera*–encoded enzymes involved in amino acid biosynthesis, and moreover the intact pathway was predicted to require the activities of enzymes encoded by both genomes. Pathway analyses predicted that enzymatic contributions from both host and symbiont genomes are needed to produce ten essential amino acids; Arg, His, Ile, Leu, Lys, Met, Phe, Try Thr, and Val (Fig. 3). While MED/Q encodes enzymes that contribute precursor substrates for Trp, Phe and Thr synthesis, *Portiera* encodes key enzymes required to complete the synthesis of these amino acids. Conversely, *Portiera* provides intermediates required by MED/Q encoded portions of the corresponding pathways that synthesize Arg, His, Ile, Leu, Lys, Met, Tyr, and Val (Fig. 3a). Our analyses of analogous amino acid biosynthesis pathways also found that not only MED/Q, but also its *Hamiltonella* endosymbiont, are fully capable of synthesizing Cys, Lys, Pro, and Thr (Additional file 25: Figure S4). Comparative genomic pathway analyses also showed that MED/Q lacks enzymes required to synthesize five B-class vitamins; biotin, folate, NAD, riboflavin, and vitamin B6, such the *Hamiltonella* genome was predicted to encode enzymes necessary for the biosynthesis of these B vitamins (Fig. 4; Additional files 26 and 27: Tables S12 and S13).

We used a phylogenetic approach to test the hypothesis that horizontal gene transfer (HGT) was involved in the gain of certain amino acid biosynthetic pathways. Our results suggest that the MED/Q genome has likely integrated genes derived from the endosymbiotic bacteria *Portiera*. The pipeline we used identified 11 putative HGT events based on phylogenetic clustering of MED/Q genes with bacterial counterparts (Additional file 28: Figure S5). In 10 of these 11 predicted HGT events, the clusters of MED/Q encoded genes were most closely related to bacterial orthologs. In the other instance, the MED/Q gene for argininosuccinate synthase was at the base of a bacterial-origin clade and adjacent to a second insect-derived clade (Additional file 28: Figure S5). Functional annotations suggest that six HGT events (those involving argH, 2 dapF paralogs, lysA, dapB, and E3.1.3.15B) are likely involved in the complementation of Arg, His, and Lys biosynthetic pathways (Fig. 3a). Six MED/Q genes involved in these 11 putative HGT events contained introns and four had a 5′-untranslated region (5′-UTR), in spite of the fact that their closest evolutionary relationships were to prokaryotic orthologs (Additional file 29: Table S14). Comparisons between amino-acid synthesis pathways in host-endosymbiont relationships (MED/Q-*Portiera*, *A. pisum*-*Buchnera* and *N. lugens*-yeast-like) suggest that MED/Q endosymbionts play an essential role in the production of seven essential amino acids (Fig. 3b). By comparison, aphid- and planthopper-endosymbiont systems primarily encode transaminases (Additional file 30: Table S15), and provide either substrates or intermediates involved in the regulation of amino acid synthesis. While aphid and planthopper endosymbionts have a role in glycolysis and the pentose phosphate pathway, *Portiera* lacks a detectable role in either pathway (Fig. 3a, c).

### Functional validation of MED/Q genes encoding detoxification enzymes

**Fig. 5:**
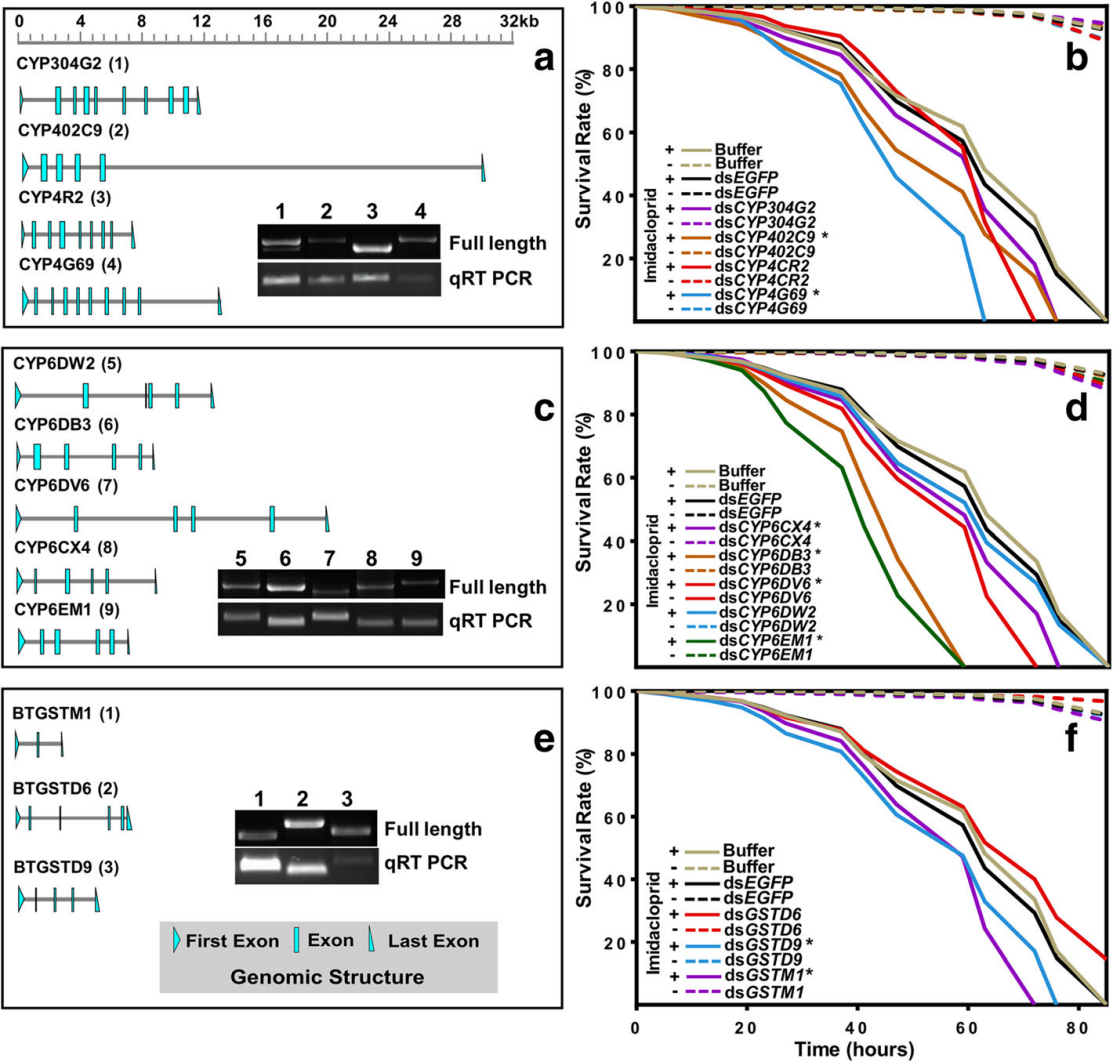
Functional validation of detoxification enzymes using dietary RNAi. The genomic structure of *CYP4s* (*CYP304G2*, *CYP402C9*, *CYP4CR2* and *CYP4G69*), *CYP6s* (*CYP6CX4*, *CYP6DB3*, *CYP6DV6*, *CYP6DW2* and *CYP6EM1*), and *GSTs* (*GSTD6*, *GSTD9* and *GSTM1*) are respectively shown in panels (**a**), (**c**), and (**e**). Images from gel electrophoresis of PCR and qRT-PCR products are displayed within the inset pictures. Plots of survival rates of *CYP4*, *CYP6*, and *GST* knockouts when exposed to 0.2 mM imidacloprid are documented in (**b**), (**d**), and (**f**), respectively (* *p* < 0.05; ** *p* < 0.01). Control treatments include non-insecticide (−, buffer, EGFP and genes), vehicle (+, buffer), and control gene (+, EGFP). Buffer are an aqueous artificial diet solution containing 5% yeast extract and 30% sucrose (wt/vol)

Nine cytochrome CYP450 (CYP304G2, CYP402C9, CYP4R2, CYP4G69, CYP6CX4, CYP6DB3, CYP6DV6, CYP6DW2 and CYP6EM1) and three GST genes (BTGSTM1, BTGSTD6 and BTGSTD9) were confirmed as being expressed in MED/Q (Fig. 5), and selected for the functional validation via RNAi knockdown. The involvement of genes encoding nine CYP450s and three GSTs in imidacloprid resistance was validated using in vivo dietary RNAi. The results showed that all of the nine P450 genes and three GST genes were significantly decreased after feeding dsRNA in 24 h, but the RNAi efficiency and the duration of effective knockdown of these genes by RNAi were different, such as for CYP402C9, CYP6DB3, CYP6DV6, CYP6EM1 and GSTD6 (Additional file 31: Figure S6).

The susceptibility of *B. tabaci* controls and RNAi knockdown individuals to 0.2-mM imidacloprid was documented. In CYP4 subfamily, the median survival rate of dsEGFP (enhanced green fluorescent protein) controls was 63%. In contrast, the survival rate of CYP4 subfamily knockouts, including CYP304G2, CYP402C9, CYP4CR2, and CYP4G69, was 63, 59, 63, and 47%, respectively (Fig. 5b). Imidacloprid bioassays showed that while the survival of *B. tabaci* CYP304G2 and CYP4CR2 RNAi knockdown individuals did not differ from the controls, survival of those from CY402C9 and CYP4G69 knockdown groups were significantly lower using Log-rank analysis. For the CYP6 subfamily experiments, the median survival rate of CYP6CX4, CYP6DB3, CYP6DV6, CYP6DW2, and CYP6EM1 knockouts was 59, 47, 59, 63, and 41%, respectively (Fig. 5d). Log-rank analysis revealed that the silencing of CYP6CX4, CYP6DB3, CYP6DV6, and CYP6EM1 significantly decreased survivorship, while silencing of CYP6DW2 did not affect survival rate. Among the GSTs that were tested, the median survival rate of GSTM1, GSTD9, and GSTD6 knockdown individuals was 59, 59, and 72% (Fig. 5f), with the corresponding imidacloprid exposed *B. tabaci* showing a significantly decrease in survivorship for groups within the GSTM1 and GSTD9 knockdown treatments.

## Discussion

Species in the *Bemisia* complex have a high reproductive rate, broad host plant range, and have adapted to a wide range of habitats. The level of standing genetic and genomic variation among *Bemisia* likely contribute to their capacity to rapidly develop insecticide resistance traits and adapt to novel habitats during biological invasions [24, 27]. The evolutionary history of *Bemisia* has likely altered the genome architecture via random processes (i.e. mutation, drift) and multiple adaptive mutations [28]. In addition, previous reports at the transcriptome level estimated that the average CDS divergence between ortholog transcripts of MEAM1/B and MED/Q was 0.83% [29], which is approximately two-fold higher than comparisons between human and chimpanzee (0.45%) [30]. While at the DNA level, the divergence is measured by dividing the number of substitutions by the number of base pairs compared for a given sequence [31]. In this study syntenic regions comprising 513 Mb were identified between MED/Q and MEAM1/B. Within these regions, 8.17% divergence was calculated (5.26% due to base substitutions and was an additional 2.91% difference due to indels), which is also approximately two-fold higher than the 5% divergence at the DNA region between human and chimpanzee [32]. Furthermore, the number of nucleotide substitutions that change amino acids (Ka) and the number of substitutions that do not (Ks) were calculated, and the ratio used as a measure to infer the impacts of directional selection on protein coding sequences [33, 34]. Among the 4052 pairs of coding sequences compared between MED/Q and MEAM1/B, the average Ka/Ks ratio is 0.236, which was closed to the estimates provide in a previous study (0.225) [29] and similar to the average Ka/Ks ratios between coding region from rat and mouse (0.19) and of human and chimpanzee (0.22) [30, 35]. Of the 2985 *B. tabaci* orthologous pairs for which Ka and Ks could be calculated, 59 have Ka/Ks > 1, suggesting the impact of positive selection on these genes. The potential adaptive significance of directional selection at these loci with respect altered gene function and impacts on speciation and adaptive evolution of the whitefly remain unknown. Predictions of functional consequences of these predicted non-synonymous changes to these genes enriched for function in DNA replication, proteasome and hematopoietic cell lineage processes were not investigated due to difficulties in doing so without detailed experimentation. More importantly, this study shows no evidence of positive selection between orthologous pairs of cytochrome P450s from MED/Q and MEAM1/B, according to the corresponding Ka/Ks values between species.

Evolutionary processes can lead to the functional diversification of duplicated gene family members, such that temporal and spatial variation results in stage- or tissue-specific gene expression patterns and derived functions [36]. When assessing copy number variation across gene family members with annotation as metabolism and detoxification both MED/Q and MEAM1/B genomes show relative parity, with the exception an increase and decrease respectively in cytochrome P450 and UGT members within MED/Q (Fig. 1a). An increased number of P450s in the CYP3 and CYP4 clades was previously shown in the genomes of *T. castaneum* genome [37], MEAM1/B [24], and analogously here for MED/Q (Fig. 1). Interestingly, our results show that the CYP3 and CYP4 gene families have undergone a further expansion in MED/Q compared to MEAM1/B. Previous experimental evidence demonstrates that both CYP3 and CYP4 gene family members function in the detoxification of xenobiotics, and are in many instances involved in the evolution of detoxification based insecticide resistance mechanisms among arthropod species [38–42]. The expansion in copy number within these detoxifying CYP3 and CYP4 gene families in MED/Q and MEAM1/B genomes might suggest that evolutionary adaptive processes in the shared histories of whitely may have selected for this phenomenon. Furthermore, a larger repertoire of P450s among *B. tabaci* might also suggest their increased breathe of xenobiotic detoxification capacity, and thus the potential to rapidly adapt to chemical insecticide exposures, and may contribute to the detoxification of host plant defenses required for a broad host range. Although circumstantial, it has been previously shown that P450 genes in *Bemisia* could be induced under various conditions, including changes in host plants, as well as being upregulated in insecticide-resistant strains [43]. Since these genes are also constitutively expressed under normal conditions [44], it is germane to suggest they are either primed for rapid response to stress or involved in cellular homeostasis. Although neonicotinoid insecticides are effective against phloem-sucking insects, including whiteflies, aphids, and thrips, high levels of resistance have been documented in the field for *B. tabaci* populations worldwide [45–47]. Based on previous studies that showed some P450s and GSTs are overexpressed in neonicotinoid-resistant *Bemisia* [44, 48–53], this study functionally validated nine CYP450s and three GSTs by RNAi-mediated knockdown. Subsequent bioassays demonstrate that the suppression of a CYP6 gene, *CYP6EM1*, significantly increased the susceptibility of *B. tabaci* to imidacloprid, indicating a potential role in the resistance mechanism for the neonicotinoid insecticides. Secondly, both MEAM1/B and MED/Q have successfully become established in invasive geographic ranges, especially in China where the introduction of MED/Q has led to the displacement of the formerly dominant invasive MEAM1/B from many localities [7, 54]. The expression level of detoxification genes was previously shown to be higher in invasive MED/Q [44], but any potential relationship with increased adaptive capacity with this invaded range remains unknown.

Endosymbiosis between host eukaryotic cells and intracellular microorganisms involves a series of adaptive changes, involving gene loss or gain resulting in complementation across one or more biosynthetic pathway. The most ancient of these is likely can be argued to be the acquisition of environmental bacteria and subsequent HGT that led to the current mitochondrion and the nuclear-encoded mitochondrial components of the ATP biosynthesis pathway. Insects contain an array of intracellular bacteria and fungi, a proportion of which have formed mutualistic relationships with their hosts; such partnership appear particularly common in the order Hemiptera [55, 56]. The impact of these symbiotic relationships on the metabolic capacity of host genomes are just starting to be revealed through whole-genome analyses [57]. All *Bemisia* species host the primary endosymbiont *Portiera aleyrodidarum* [58] and an array of species-specific secondary endosymbionts: *Arsenophonus* spp. (*Enterobacteriales*), *Wolbachia* spp. (*Rickettsiales*), “*Candidatus* Hamiltonella defensa” (*Enterobacteriales*), “*Candidatus* Hemipteriphilus asiaticus”, “*Candidatus* Cardinium hertigii” (*Bacteroidales*), “*Candidatus* Fritschea bemisiae” (*Chlamydiales*) and *Rickettsia* spp. (*Rickettsiales*) [59–62]. Previous studies have suggested the potential for the metabolic complementarity between *Bemisia* and *Portiera*, and between *Bemisia* and *Hamiltonella* [16, 17]. Transcriptome-based analysis of MEAM1/B that compared both bacteriocytes and the whole-body samples reported that the host genome may contribute enzymes that complement or duplicate *Portiera*–encoded pathways, and that *Hamiltonella* might contribute multiple cofactors and the pathway for producing the essential amino acid Lys [16]. Analysis of *Portiera* genome predicted a lack of genes essential for the biosynthesis of certain amino acids, which was supported by congruent findings from a transcriptome-based approach [13, 63]. Our work provides the first assessment of gene reduction and metabolic pathway complementation in MED/Q-symbiont relationships. Our comparative genome approach suggests that *Portiera* encodes pathways that synthesize of 10 essential amino acids (Fig. 3) and that *Hamiltonella* contributes B-vitamins (Fig. 4), none of which can be produced by MED/Q, the host along. The metabolic/nutritional contributions of *Portiera* and *Hamiltonella* to MED/Q allow host to survive/thrive on nutrient deficient diets (phloem sap).

The presence of numerous metabolic pathways that require gene products from both the host and endosymbionts suggests a strong obligate symbiosis. Such complementarity likely could evolve through gene reduction (loss) in either the host or endosymbionts following a relaxation of selective constraints when redundant copies are present, or can also occur through HGT. For instance, enzymes involved in carotenoid biosynthesis were derived from fungal genes integrated into the *A. pisum* genome [64]. HGT in the citrus mealybug, *Planococcus citri*, also contribute to functions absent in its endosymbionts, which evolved a different system of amino acid synthesis [65]. The fact that enzymes require from both MED/Q and *Portiera* in order to attain functional 11 different biosynthetic pathways suggests that pathway complementarity may be an initial step in the development of obligatory endosymbiotic relationships, or a consequence of the random evolutionary events that created an inseparable and co-dependent metabolic system.

Modifications to the insect immune system are necessity to facilitate the residency of previously free-living extracellular bacteria, where the host no longer recognized the bacteria as pathogenic agents. In contrast, the acceptance of foreign bacteria by the host, or evasion of host defenses by the bacteria, is essential for any intracellular symbiosis. Normal infections of *D. melanogaster* by Gram-negative bacteria activates the IMD pathway [66]. Since the obligate endosymbionts of both *Bemisia* and *A. pisum* are Gram-negative bacteria [66, 67], the loss of IMD pathway components in MED/Q and MEAM1/B genomes may be more than coincidental. The loss of IMD pathway is predicted to facilitate the acquisition of endosymbiotic bacteria [68]. We estimated that the divergence of *Bemisia*, *A. pisum*, *D. citri*, and *N. lugens* occurred 286 million years ago (MYA) (Additional file 11: Figure S2), which is consistent with other research dating the divergence of Auchenorrhyncha (containing *N. lugens*) and Sternorrhyncha (containing *A. pisum*, *Bemisia,* and *phylloxera*) to 290 MYA [69]. These evolutionary events and recent studies suggest that the ancestors of *Bemisia* and *A. pisum* acquired their obligate endosymbionts after separating from the ancestral group containing *N. lugens*, a split that may have involved a loss-of-function event in the IMD pathway of the former group. The absence of a functioning IMD pathway in MED/Q may also have contributed to the subsequent acquisition of *Hamiltonella* and other facultative endosymbionts that have been detected in members of the *Bemisia* complex, but additional research into the diverse aspects of these relationships is required.

## Conclusions

The expansion of detoxification genes families like P450s are observed in *B. tabaci* MED/Q and MEAM1/B genomes, which is suggestive of an evolutionary-driven adaptive response in the history of the whitefly lineage. Regardless of these unknown past events, the larger repertoire of detoxification functionalities among variant P450 might suggest an increased capacity to respond to and survive exposures to chemical insecticides as well as xenobiotic host plant defenses. Both of these aspects may further facilitate the invasive abilities observed for MED/Q and MEAM1/B, whereby both have invaded novel geographic ranges and ecological niches. In support of this hypothesis that the expansion of metabolic resistance genes in the MED/Q genome could contribute to observed chemical insecticide resistance traits in the population, conventional gene expression analysis and RNAi-mediated functional validation demonstrated the role of P450s in *B. tabaci* resistance to the neonicotinoid insecticide, imidacloprid. The analysis of *Bemisia* MED/Q genome also uncovered the biochemical basis for the nutrient/nutritional partitioning between MED/Q host and endosymbionts, which involves the complementation and horizontal transfer of pathway components (enzymes) between genomes. A reduction in the IMD immune system in MED/Q also suggests host adaptations that lead to acceptance of intracellular bacterial may be a key process that likely has facilitated the development of the relationship. of *Bemisia* and their symbionts. More importantly, this MED/Q genomic resource provides a foundation for future ‘pan-genomic’ comparisons of across the cryptic *Bemisia* spp., such as between invasive vs. non-invasive, invasive vs invasive, and native vs. exotic strain. Undoubtedly, the current genomic resources will likely open new avenues of research into whitefly biology, ecology and evolution, and could facilitate the development of new strategies for the management of this severe agricultural species.

## Methods

### Identification and analysis of the orthologous genes

Data from two recently published *B.tabaci* genomes data set, MEAM1/B, http://www.whiteflygenomics.org/cgi-bin/bta/index.cgi, v1, [24] and MED/Q, http://gigadb.org/dataset/view/id/100286/token/etFfO6xzVU8Iv5Kk [24] were downloaded. In order to study the syntenic blocks between these two sequences, an alignment of MED/Q and MEAD1/B draft assemblies in fasta format was performed using LastZ [26]. To compare the level of coding sequence divergence between these two species, we analyzed the gene coding sequence variance between possible orthologous genes identified using *TreeFam* [70]. This involved as following steps, which has been widely used to identify orthologous genes: Firstly, protein sequences of these two species were compared by *blast* with the *E*-value threshold 1e-7. Secondly, high-scoring segment pairs (HSPs) of each protein pair were concatenated by *solar* [71]. H-scores were computed based on Bit-scores and used to evaluate the similarity among genes. Finally, gene family members were predicted by clustering of homologous gene sequences using *Hcluster_sg* (version 0.5.0) [72, 73]. To compare the genomics difference among MED/Q and other insects, we further collected 13 other insects and 1 crustacean genome data sets: *A. pisum, R. prolixus, B. mori, D. plexippus, N. vitripennis, T. castaneum, P. humanus, D. melanogaster, A. gambiae,* and *D. pulex* (ftp.ensemblgenomes.org/); *A. mellifera, N. lugens,C. floridanus* and *D. citri* (ftp.ncbi.nih.gov). Then we identified and analyzed their orthologous genes using above methods.

### Phylogenetic tree reconstruction and divergence time estimation

The coding sequences of single-copy gene families conserved among MED/Q and other 15 species were extracted, translated into amino acid sequences, and aligned by the program MUSCLE (MUSCLE, RRID:SCR_011812) [74]. The individual sequence alignments were then concatenated into one supermatrix. PhyML (PhyML, RRID:SCR_014629) [75, 76] was applied to construct the phylogenetic tree under a GTR + gamma model for nucleotide sequence evolution. aLRT values were taken to assess the branch reliability in PhyML. The same set of codon sequences at position 1 was used for phylogenetic tree construction and estimation of the divergence time. The PAML mcmctree program (PAML, RRID:SCR_014932) (PAML version 4.5) [77, 78] was used to determine divergence times with the approximate likelihood calculation method and the correlated molecular clock and REV substitution model.

### Detection of positively selected genes and fast-evolving genes

We calculated Ka/Ks ratios for all single copy orthologs of five phloem feeding insects (*B. tabaci* MED/Q and MEAM1/B, *A. pisum*, *N. lugens* and *D. citri*). For the CDS region, pair-wise alignments were generated for the single copy orthologous gene pairs based upon translated protein sequences, and then back translated to DNA sequences for subsequent analysis. Alignment quality was essential for estimating purifying/fast evolving genes and positively selected genes. Thus orthologous genes were first aligned by PRANK [79], which is considerably conservative for inferring positive selection. *Gblocks* [80] was used to remove ambiguously aligned blocks within *PRANK* alignments and employed ‘codeml’ in the PAML package with the free-ratio model to estimate Ka, Ks, and Ka/Ks ratios on different branches. The differences in mean Ka/Ks ratios for single-copy genes between MED/Q and each of the other species were compared using paired Wilcoxon rank sum tests. Genes that showed values of Ka/Ks higher than 1 along the branch leading to MED/Q were reanalyzed using the codon based branch-site tests implemented in PAML (PAML, RRID:SCR_014932). The branch-site model allowed ω to vary both among sites in the protein and across branches, and was used to detect episodic positive selection. To detect the fast-evolving genes between MED/Q and MEAM1/B, we employed ‘*KaksCaculator*’ with YN model to estimate Ka, Ks, and Ka/Ks ratios between gene pairs.

### Gene family expansion and contraction

Gene family expansion and contraction analysis were performed using the software *CAFE 2.1*. In CAFE [81], a random birth and death model was used to predict gene gain and loss among gene families across the species-specific phylogenetic tree. Fisher’s exact test (*p*-value < 0.01) was used to test for over-represented functional categories (GO terms) among the expanded genes and the remainder of non-expanded genes across the genome.

Detoxification enzymes within the putatively expanded cytochrome P450 monooxygenase (CYP450) gene family were identified using a homology-based strategy that used reference *D. melanogaster*, *A. pisum*, *A. gambiae*, and *A. mellifera* gene models downloaded from (NCBI, http://www.ncbi.nlm.nih.gov/). First, we identified the detoxifying enzyme genes in MED/Q gene models by querying our gene set and scaffolds data with orthologous sequences using the BLASTx algorithm (*E*-value ≤10^− 5^). The genomics segments with hits were linked by the Solar software, and parsed using Genewise software for gene predictions to enable the identification of full-length coding sequences. The resultant sequences were filtered in searches against the non-redundant (nr) and Interpro databases. After filtering false-positive matching sequences, the genes were manually corrected using the MED/Q transcriptome (mainly to P450 and UGT manual annotation), and phylogenetic trees were constructed using MEGA6.0 [82]. A similar method was applied to identify homologous genes in other selected insects.

The immunity related genes in MED/Q were identified by combining the results from motif- and homology-based strategies, as previously described [83]. This comparison was accomplished by downloading the query sequences available in ImmunoDB [84], and from the NCBI database for the six insects *D. melanogaster*, *A. gambiae*, *A. aegypt*, *A. mellifera*, *C. quinquefasciatus* and *A. pisum*. For the motif-based search, MAFFT [85] was used to align multiple protein sequences followed by the software HMMER 3.0 that was used to build models against which MED/Q sequences, and these models were used a queries to searched downloaded sequence databases using tBLASTn with hits linked using the Solar software. Genewise software [86] was used to improve the gene predictions and to obtain full-length gene sequences. The resultant MED/Q sequences were edited manually and merged into a combined dataset of putative immune-related genes. The immune-related genes of *A. pisum* and *N. lugens* were used for comparisons with whitefly genes obtained using a similar approach.

### In silico metagenomic analysis of MED/Q endosymbiosis

To improve the *B. tabaci* MED/Q-*associated Hamiltonella* draft genome (AJLH00000000), sequencing data from 16 Illumina paired-end read libraries, ranging from 170 bp to 40 kb (44 lanes), from the MED/Q genome-sequencing project were used. Four sequences were selected as references to filter candidate reads using SOAPaligner (Version: 2.21). These sequences included a previous draft of the *Hamiltonella* genome (372 scaffolds; AJLH00000000), the pea aphid *Hamiltonella* genome (CP001277), the *Yersinia pestis* CO92 complete genome (AL590842), and the *Serratia plymuthica* AS9 genome (CP002773). The SOAPaligner parameters were “-v 5” for the short insert size library (< 1 kb) data and “-v 3 -R” for the large insert size library (> 1 kb) data. SOAPdenovo (version 2.04) was used for genome assembly, using the parameters “-u -d 1 -F -K 45” on the above 170 bp to 40 k bp data. Gap filling was performed after scaffold construction, and a super-scaffold was obtained using the paired-end reads on > 500 bp scaffolds to reduce the scaffold number. Then, the Unique Genome Profile (UGP) pipeline was applied to link the scaffolds using BAC sequences from MED/Q. Briefly, 1) the flanking 20 kb sequences of each scaffold were removed, and then unique tags (31-mer) were constructed; 2) the BAC sequences (< 150 kb) were used as queries in BLASTn searches against the unique genomic tags; and 3) BACs that had more than two hits were filtered, and then used to construct link relationships to connect larger scaffolds. In addition, the genome of the MED/Q-associated primary endosymbiont *Portiera* was filtered and assembled (as described above) together with four previously reported *Portiera* genome reference sequences obtained from *B. tabaci* B and Q genome (GenBank: CP003708, CP003868, CP003867 and CP003835).

Genes were predicted for the finished *Hamiltonella* and *Portiera* genomes using Glimmer v3.02 (protein-coding genes), tRNAscan-SE (tRNAs) and RNAmmer v1.2 (rRNA). The putative coding sequences were annotated using BLASYp similarity searches that showed consensus to the NR database (20121005). The *E*-value cutoff ≤10^− 5^ and a minimum match percentage of 40% were used to filter results. Protein domain searches were conducted using InterProScan v4.8, available at the Pfam database, and the resulting coding sequences were used to search the KEGG database (http://www.genome.jp/tools/kaas/).

The amino acid synthesis-related genes in the MED/Q genome were searched against the NR database, under the scenario that they were not of insect origin. The genes identified in this way were used to construct a phylogenetic tree. To confirm that the HTGs identified were not contaminants associated with bacterial sequences in the libraries, hits were required to satisfy at least one of the two following conditions: 1) the HTGs were located on scaffolds that included coding regions homologous to other insects; and 2) the HTGs’ transcripts should be present in alignments to a current transcriptome database (after manual corrections) and also as corresponding genes encoded by the genome.

### Quantitative real-time PCR

Total RNAs were extracted from 30 to 40 *B. tabaci* adults (mixed sexes, female: male = 1: 1) per strain using a TRIzol reagent following the manufacturer’s protocol (Thermo-Fisher, Wilmington, DE, USA). The total RNA was resuspended in the nuclease-free water and quantified with a Nanodrop 2000 spectrophotometer (Thermo-Fisher, Wilmington, DE, USA). Subsequently, the first-strand cDNAs were synthesized using the PrimeScript® RT reagent Kit (Takara Biotech, Tokyo, Japan) with gDNA Eraser according to the manufacturer’s protocol. Reverse transcription was performed on 1.0 μg of each RNA sample. Synthesis of P450 and GST genes dsRNA and application of RNAi to insect were carried out according to published protocols. In addition, dsRNAs were prepared using the T7 RiboMAX Express RNAi system and protocols (Promega, Madison, WI, USA).

A total 120 adults (three biological replicates, *n* = 40) were subjected to qRT-PCR analysis. Nine cytochrome CYP450 and three GST sequences were selected from this MED/Q genomic sequencing project. Full length primers were designed to analysis the quality of genomic genes annotation, and qRT-PCR primers were designed to amplify a 85- to 250-bp fragment at annealing temperature of 60 °C (Additional file 32: Table S16). The amplification efficiency of these q-PCR primers are 95–105%. The 20-μl reaction mixture consisted of 1.0-μl of 300-ng cDNA (3 times diluted), 10.0-μl of the SYBR® Green Real-time PCR Master Mix with ROX (Thermo-Fisher, Wilmington, DE, USA), and 0.6-μl of each primer. qPCR was conducted with the ABI 7500 system by the following protocol: 15 min of activation at 95 °C followed by 40 cycles of 10 s at 95 °C, 30 s at 60 °C, and 30s at 72 °C. A 3-fold dilution series of the whitefly cDNA was constructed the relative standard curve to calculate the amplification efficiency of the 12 genes. Elongation factor 1 alpha subunit (EF1-α) of *B. tabaci* were used as reference gene [87]. The RNA interference efficiency in CYP450 and GST genes expression, normalized to reference gene, were calculated using the 2^-△△Ct^ method [88].

### In vivo dietary RNA interference in *B. tabaci*

Dietary RNAi was carried out in a feeding chamber consisting of a glass tube (20 mm in diameter × 50 mm long that was open at both ends), which was covered at the top end by one layer of Parafilm membrane (Alcan Packaging, Chicago, IL, USA) [49]. A 0.2-mL portion of diet solution containing 5% yeast extract and 30% sucrose (wt/vol) was placed on the outer surface of the Parafilm and was covered with another layer to encase the solution between the Parafilms. Whiteflies were released into the other end of the tube. Then the tube was sealed with a black cotton plug and covered with a shade cloth. The end of the tube with a Parafilm membrane was facing a light source that was approximately 20-cm away. This dietary RNAi system was used to investigate the impact of detoxification enzymes on the susceptibility of adult *B. tabaci* to imidacloprid insecticide. Control treatments included non-insecticide (−, buffer, EGFP and genes), vehicle (+, buffer), and control gene (+, EGFP). Buffer was an aqueous solution of artificial diet containing 5% yeast extract and 30% sucrose (wt/vol). Treatments used the same artificial diet solution mixed with dsRNAs of P450s and GSTs (0.5-μg/μL) [49]. dsRNAs were synthesized in vitro using a T7 RiboMAX Express RNAi system following the manufacturer’s protocol (Promega, P1700, USA). In the insecticide toxicity assay, *B. tabaci* adults were fed on both treatment and control diets containing 0.2-mM imidacloprid. Mortality pt?>was assessed after 2 h of feeding. Newly emerged adults (24 h within hatching, mix sexed) were introduced into the feeding chamber, and placed in an environmental chamber at 25 °C, a photoperiod of L14: D10, and 80% RH. Survival data were analyzed with log-rank test using SPSS (SPSS for Windows, Rel. 17.0.0 2009. Chicago: SPSS Inc.).

## Additional files

Additional file 1: Annotation of the unique genes from MED/Q genome relative to MEAM1/B. (XLS 904 kb)

Additional file 2: Annotation of the unique genes from MEAM1/B genome relative to MED/Q. (XLS 1500 kb)

Additional file 3: Table S1. Reads mapped ratio of MED/Q and MEAM1/B with each other. (DOCX 13 kb)

Additional file 4: Table S2. Syntenic alignment between MED/Q and MEAM1/B genome. (DOCX 16 kb)

Additional file 5: Figure S1. Syntenic blocks between *Bemisia tabaci* MED/Q and MEAM1/B genome. (TIFF 10551 kb)

Additional file 6: Table S3. Identification and analysis of the orthologous genes between MED/Q and MEAM1/B. (DOCX 15 kb)

Additional file 7: Orthologs with existed both Ka and Ks. (XLS 576 kb)

Additional file 8: Orthologous with Ka/Ks value larger than 1. (XLS 6 kb)

Additional file 9: GO enriched results of the sequences between MED/Q and MEAM1/B with Ka/Ks values > 1. (XLSX 16 kb)

Additional file 10: KEGG enriched results of the sequences between MED/Q and MEAM1/B with Ka/Ks values > 1. (XLSX 17 kb)

Additional file 11: Figure S2. Estimated divergence times among insect genomes using PAML *mcmctree*. (TIFF 646 kb)

Additional file 12: Orthologous gene families specific from MEAM1/B in the MED/Q. (XLS 57 kb)

Additional file 13: Orthologous gene families specific from others in the MED/Q. (XLS 29 kb)

Additional file 14: Table S4. Gene ontology of gene families specific in MED/Q from MEAM1/B (FDR < 0.05). (DOCX 51 kb)

Additional file 15: Table S5. Gene ontology of gene families specific in MED/Q from other 15 species (FDR < 0.05). (DOCX 51 kb)

Additional file 16: Candidate PSGs in MED/Q. (XLS 13 kb)

Additional file 17: Candidate PSGs in MED/Q and MEAM1/B. (XLS 7 kb)

Additional file 18: Table S6. Gene ontology of PSG in MED/Q and MEAM1/B (FDR < 0.05, *P* < 0.01). (DOCX 51 kb)

Additional file 19: Table S7. Gene ontologies for gene families that have expanded number of members on MED/Q branch (FDR < 0.05, *p* < =0.000515776699029126). (DOCX 50 kb)

Additional file 20: Figure S3. Gene family expansion and contraction in *B. tabaci* Q genome compared to other arthropods. (TIFF 141 kb)

Additional file 21: Table S8. Gene ontologies for gene families that have expanded number of members on *Bemisia tabaci* branch (FDR < 0.05, *p* < =0.000879854368932039). (DOCX 53 kb)

Additional file 22: Table S9. Gene ontology over-representation of gene families contracted on *Bemisia tabaci* branch (FDR < 0.05, *p* < =0.000572390572). (DOCX 49 kb)

Additional file 23: Table S10. Gene ontology over-representation of gene families contracted on *Bemisia tabaci* branch (FDR < 0.05, *p* < =0.000572390572). (DOCX 49 kb)

Additional file 24: Table S11. Immune system-related and virus transport genes in phloem- and blood-feeding insects. (DOCX 56 kb)

Additional file 25: Figure S4. Pathways encoded by *Candidatus hamiltonella* for amino acid biosynthesis. The major components of amino acid pathway encoded by *Hamiltonella*, a facultative *Bemisia* endosymbiont (essential amino acids in pink, unessential amino acids in black). *Hamiltonella* genes are highlighted in blue boxes with names corresponding to its genome (PRJNA299727), while white boxes indicate genes that do not have a match in MED/Q genome or *Hamiltonella* genome. (PNG 52 kb)

Additional file 26: Table S12. Genes involved in B vitamin biosynthesis in MED/Q. (DOCX 50 kb)

Additional file 27: Table S13. Genes involved in B vitamin biosynthesis in *Candidatus Hamiltonella* defense. (DOCX 51 kb)

Additional file 28: Figure S5. Phylogenetic trees for 11 horizontally transferred genes (HGTs). (TIFF 8589 kb)

Additional file 29: Table S14. Horizontally transferred genes involved in amino acid biosynthesis in MED/Q. (DOCX 50 kb)

Additional file 30: Table S15. Comparison of transaminases in three symbiotic systems *Bemisia tabaci*/Portiera, *Acyrthosiphum pisum*/Buchnera and *Nilaparvata lugens*/Yeast-like. (DOCX 50 kb)

Additional file 31: Figure S6. The RNA interference efficiency of nine CYP450 and three GST gene. mRNA levels of these genes were quantified by qRT-PCR in 96 h of feeding on a diet containing dsEGFP and dsCYP450/dsGST. The mRNA levels are shown as a ratio relative to the levels for the reference gene (EF1α). Values are means ± SEMs (*n* = 3, * *p* < 0.05; ** *p* < 0.01, two-tailed Student’s t-test). (TIFF 1068 kb)

Additional file 32: Table S16. Primers used in this study. (DOCX 20 kb)

## Abbreviations

COE: Carboxyl/choline esterases
GST: Glutathione S-transferases
HTGs: Horizontally transferred genes
IMD: Immune deficiency
MED/Q: Mediterranean *Bemisia tabaci* Q
MEAM1/B: Whitefly *Bemisia tabaci* MEAM1 or Whitefly *Bemisia tabaci*B
MYA: Million years ago
P450: Cytochrome P450 mono-oxygenases
UGTs: UDP glycosyltransferases
