# Supplementary material for: The invasive MED/Q Bemisia tabaci genome: a tale of gene loss and gene gain
Source: BMC Genomics. 2018 Jan 22;19:68. doi: 10.1186/s12864-018-4448-9 (PMC5778671; doi:10.1186/s12864-018-4448-9)
Supplement: Supplementary file 3 — Reads mapped ratio of MED/Q and MEAM1/B with each other. (DOCX 13 kb) [file 12864_2018_4448_MOESM3_ESM.docx]

Table S1 Reads mapped ratio of MED/Q and MEAM1/B with each other.

| **Species** | **Insert size** | **Paired alignment ratio** | **Singled alignment ratio** |
| --- | --- | --- | --- |
| MED/Q | 500bp | 69.55% | 9.65% |
| MEAM1/B | 500bp | 26.63% | 30.04% |

Note: By SOAPaligner/soap2.
