# Supplementary material for: The invasive MED/Q Bemisia tabaci genome: a tale of gene loss and gene gain
Source: BMC Genomics. 2018 Jan 22;19:68. doi: 10.1186/s12864-018-4448-9 (PMC5778671; doi:10.1186/s12864-018-4448-9)
Supplement: Supplementary file 4 — Syntenic alignment between MED/Q and MEAM1/B genome. (DOCX 16 kb) [file 12864_2018_4448_MOESM4_ESM.docx]

Table S2 Syntenic alignment between MED/Q and MEAM1/B genome.

| **Species** | **Genome Size** | **Length^*^, nt** | **Length^§^, %** | **Substitution^†¶^, %** | **Indels^‡ ¶^, %** | **Total^¶^, %** |
| --- | --- | --- | --- | --- | --- | --- |
| MED/Q | 658Mb | 513,081,028 | 77.94 | 5.26 | 2.91 | 8.17 |
| MEAM1/B | 615Mb | 511,842,204 | 83.22 | 5.27 | 2.91 | 8.19 |

*The length of the region compared.

§The percentage of the compared region in genome.

†The percent of nucleotides replaced by a different nucleotide.

‡The sum of the length of all gaps in both MED/Q and MEAM1/B sequences as percent of aligned MED/Q or MEAM1/B length.

¶Weighted (by length) average.
