# Supplementary material for: The invasive MED/Q Bemisia tabaci genome: a tale of gene loss and gene gain
Source: BMC Genomics. 2018 Jan 22;19:68. doi: 10.1186/s12864-018-4448-9 (PMC5778671; doi:10.1186/s12864-018-4448-9)
Supplement: Supplementary file 6 — Identification and analysis of the orthologous genes between MED/Q and MEAM1/B. (DOCX 15 kb) [file 12864_2018_4448_MOESM6_ESM.docx]

Table S3 Identification and analysis of the orthologous genes between MED/Q and MEAM1/B

| **Species** | **#Total genes** | **#Unclustered genes** | **#Families** | **#Unique families** | **Ave. genes per family** |
| --- | --- | --- | --- | --- | --- |
| MEAM1/B | 15,664 | 3,494 | 7,202 | 158 | 1.69 |
| MED/Q | 20,748 | 4,731 | 7,794 | 750 | 2.06 |
