# Supplementary figures and images for: The invasive MED/Q Bemisia tabaci genome: a tale of gene loss and gene gain
Source: BMC Genomics. 2018 Jan 22;19:68. doi: 10.1186/s12864-018-4448-9 (PMC5778671; doi:10.1186/s12864-018-4448-9)

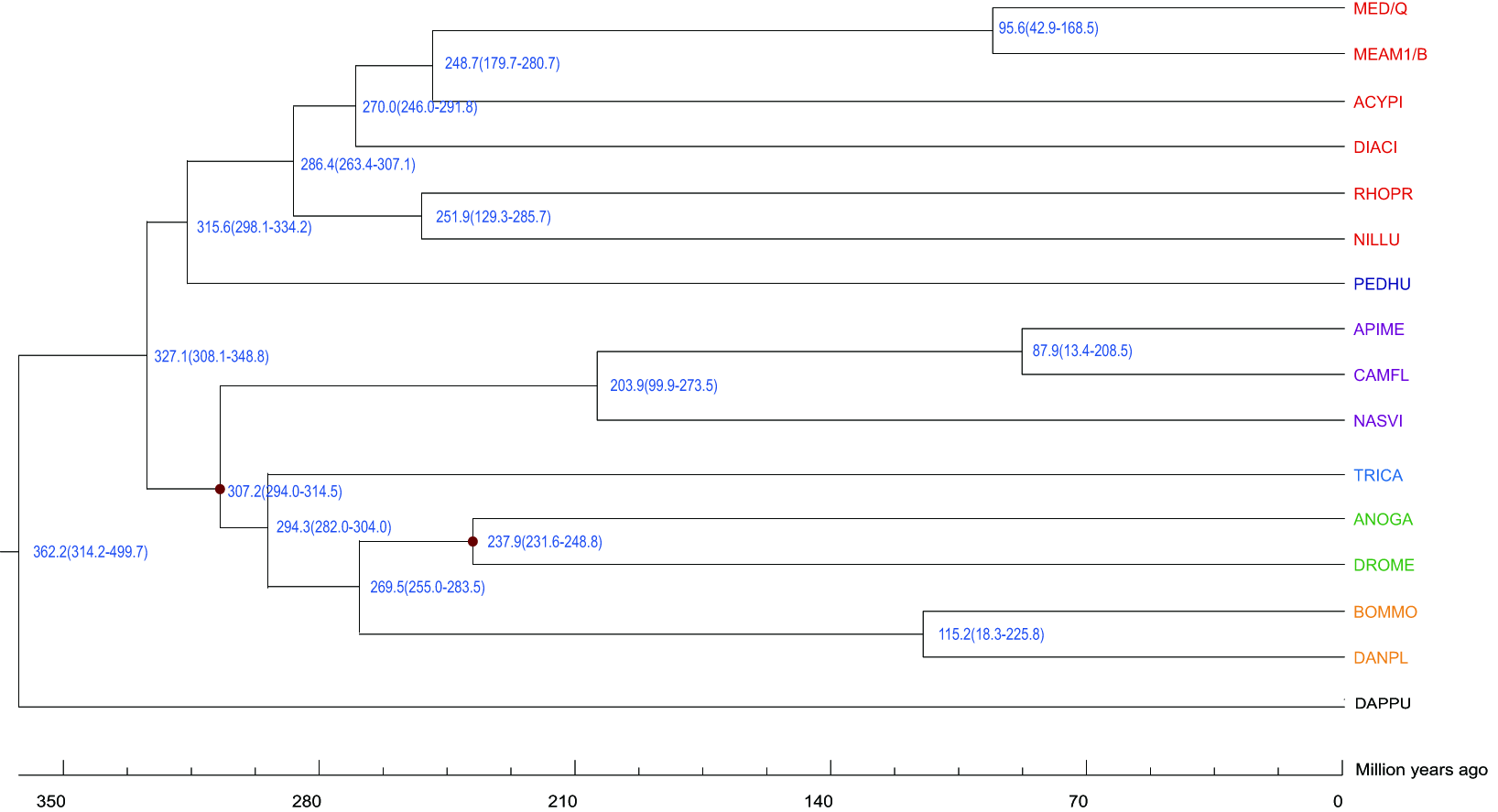

Supplement: Supplementary file 11 — Estimated divergence times among insect genomes using PAML mcmctree. (TIFF 646 kb) [file 12864_2018_4448_MOESM11_ESM.tif]

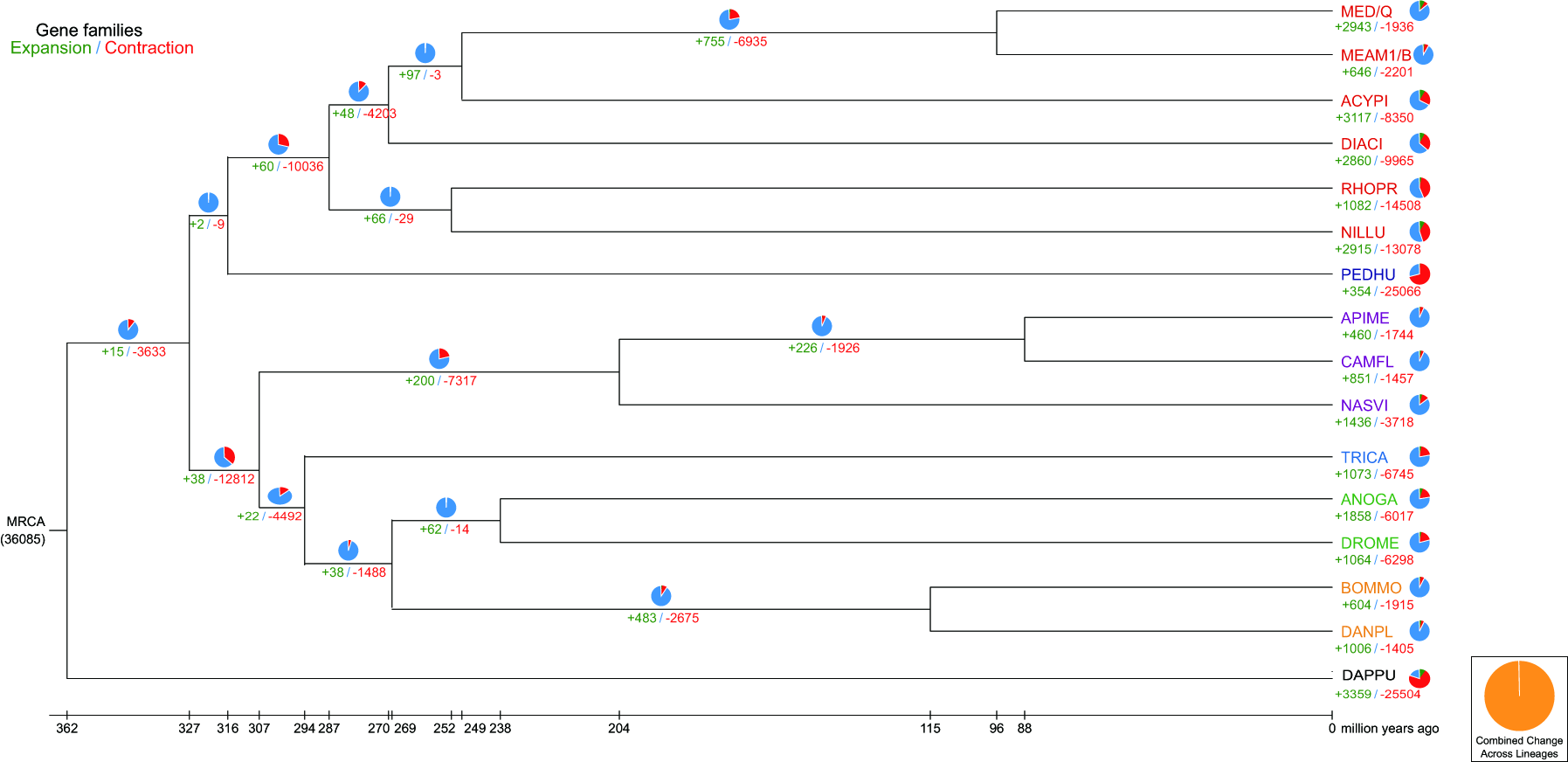

Supplement: Supplementary file 20 — Gene family expansion and contraction in B. tabaci Q genome compared to other arthropods. (TIFF 141 kb) [file 12864_2018_4448_MOESM20_ESM.tif]

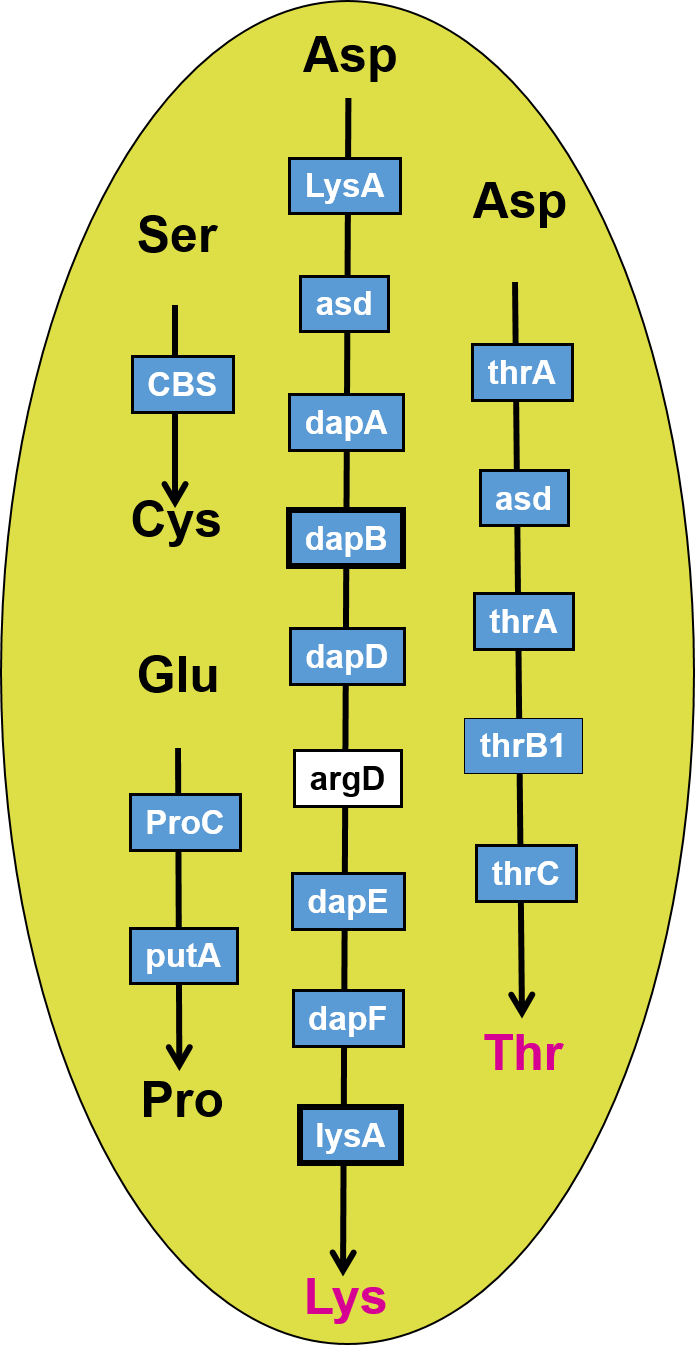

Supplement: Supplementary file 25 — Pathways encoded by Candidatus hamiltonella for amino acid biosynthesis. The major components of amino acid pathway encoded by Hamiltonella, a facultative Bemisia endosymbiont (essential amino acids in pink, unessential amino acids in black). Hamiltonella genes are highlighted in blue boxes with names corresponding to its genome (PRJNA299727), while white boxes indicate genes that do not have a match in MED/Q genome or Hamiltonella genome. (PNG 52 kb) [file 12864_2018_4448_MOESM25_ESM.png]

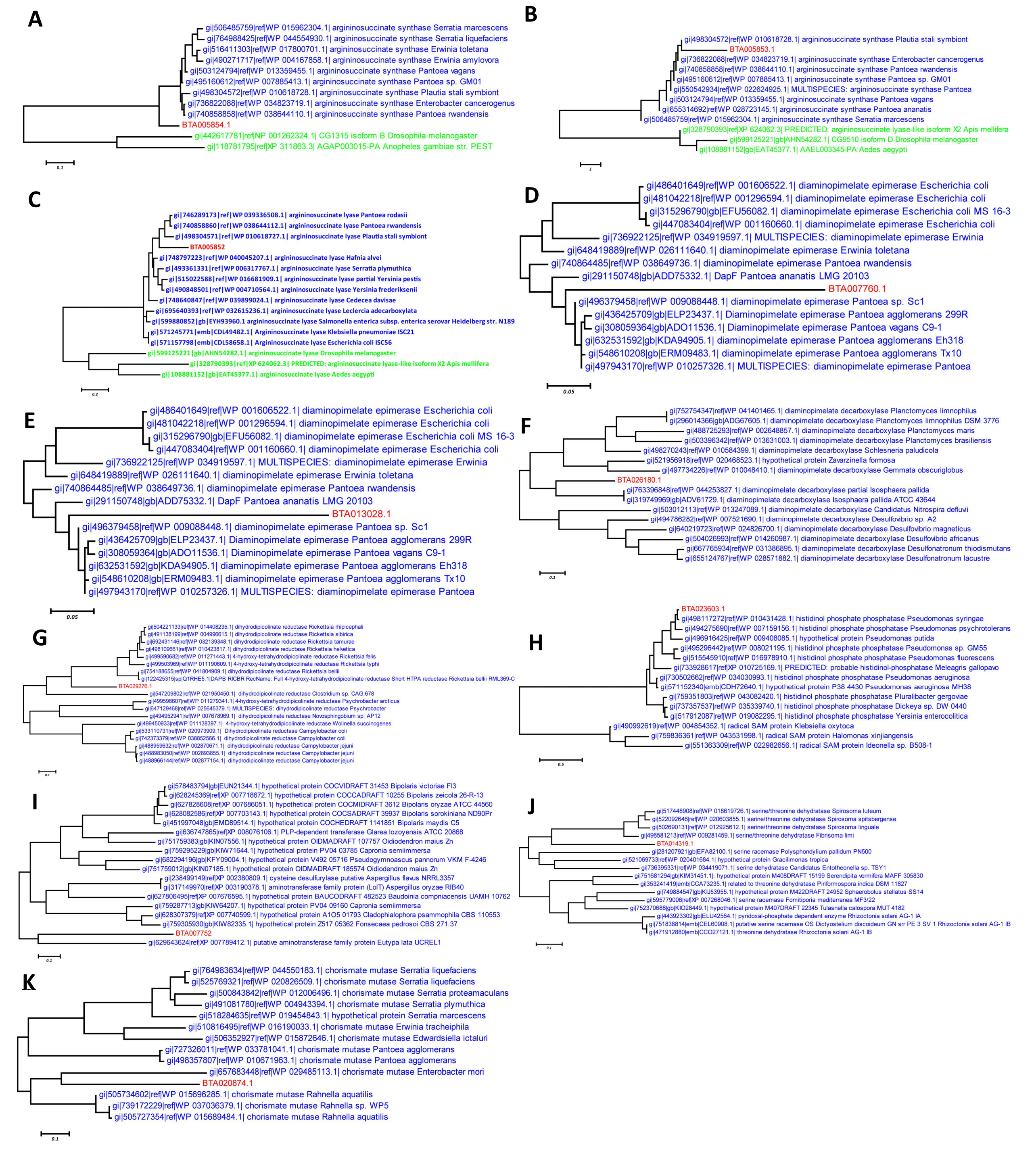

Supplement: Supplementary file 28 — Phylogenetic trees for 11 horizontally transferred genes (HGTs). (TIFF 8589 kb) [file 12864_2018_4448_MOESM28_ESM.tif]
