# Supplementary material for: The invasive MED/Q Bemisia tabaci genome: a tale of gene loss and gene gain
Source: BMC Genomics. 2018 Jan 22;19:68. doi: 10.1186/s12864-018-4448-9 (PMC5778671; doi:10.1186/s12864-018-4448-9)
Supplement: Supplementary file 14 — Gene ontology of gene families specific in MED/Q from MEAM1/B (FDR < 0.05). (DOCX 51 kb) [file 12864_2018_4448_MOESM14_ESM.docx]

**Table S4. Gene ontology of gene families specific in MED/Q from MEAM1/B (FDR<0.05)**

| **GO ID** | **GO description** | **Type** | **Number of genes** | **P-value** |
| --- | --- | --- | --- | --- |
| GO:0005215 | transporter activity | MF | 125 | 0 |
| GO:0003700 | sequence-specific DNA binding transcription factor activity | MF | 110 | 0 |
| GO:0016491 | oxidoreductase activity | MF | 94 | 2.97E-06 |
| GO:0043565 | sequence-specific DNA binding | MF | 61 | 2.82E-07 |
| GO:0004872 | receptor activity | MF | 60 | 8.37E-29 |
| GO:0000156 | two-component response regulator activity | MF | 54 | 1.15E-33 |
| GO:0000155 | two-component sensor activity | MF | 37 | 1.93E-21 |
| GO:0016887 | ATPase activity | MF | 32 | 4.76E-05 |
| GO:0004673 | protein histidine kinase activity | MF | 28 | 1.74E-18 |
| GO:0016772 | transferase activity, transferring phosphorus-containing groups | MF | 23 | 9.05E-13 |
| GO:0016769 | transferase activity, transferring nitrogenous groups | MF | 17 | 5.31E-05 |
| GO:0004871 | signal transducer activity | MF | 17 | 0.000407 |
| GO:0050662 | coenzyme binding | MF | 14 | 2.30E-05 |
| GO:0008137 | NADH dehydrogenase (ubiquinone) activity | MF | 10 | 0.000203 |
| GO:0004560 | alpha-L-fucosidase activity | MF | 7 | 0.000398 |
| GO:0008658 | penicillin binding | MF | 5 | 0.000825 |
| GO:0004887 | thyroid hormone receptor activity | MF | 5 | 0.000266 |
| GO:0016020 | membrane | CC | 208 | 1.42E-11 |
| GO:0006355 | regulation of transcription, DNA-dependent | BP | 161 | 5.77E-12 |
| GO:0006810 | transport | BP | 146 | 0 |
| GO:0000160 | two-component signal transduction system (phosphorelay) | BP | 63 | 8.30E-39 |
| GO:0009058 | biosynthetic process | BP | 44 | 1.41E-06 |
| GO:0018106 | peptidyl-histidine phosphorylation | BP | 28 | 1.74E-18 |
| GO:0016310 | phosphorylation | BP | 23 | 1.13E-14 |
| GO:0008652 | cellular amino acid biosynthetic process | BP | 11 | 2.71E-07 |
| GO:0042773 | ATP synthesis coupled electron transport | BP | 10 | 2.38E-08 |
| GO:0019318 | hexose metabolic process | BP | 5 | 5.01E-05 |
| GO:0009273 | peptidoglycan-based cell wall biogenesis | BP | 5 | 0.000266 |

Abbreviation: BP (Biological Process), CC (Cellular Component), MF (Molecular Function).
