# Supplementary material for: The invasive MED/Q Bemisia tabaci genome: a tale of gene loss and gene gain
Source: BMC Genomics. 2018 Jan 22;19:68. doi: 10.1186/s12864-018-4448-9 (PMC5778671; doi:10.1186/s12864-018-4448-9)
Supplement: Supplementary file 15 — Gene ontology of gene families specific in MED/Q from other 15 species (FDR < 0.05). (DOCX 51 kb) [file 12864_2018_4448_MOESM15_ESM.docx]

**Table S5. Gene ontology of gene families specific in MED/Q from other 15 species (FDR<0.05)**

| **GO ID** | **GO description** | **Type** | **Number of genes** | **P-value** |
| --- | --- | --- | --- | --- |
| GO:0005215 | transporter activity | MF | 101 | 4.76E-13 |
| GO:0004872 | receptor activity | MF | 59 | 2.88E-49 |
| GO:0016491 | oxidoreductase activity | MF | 46 | 4.81E-05 |
| GO:0003700 | sequence-specific DNA binding transcription factor activity | MF | 41 | 1.28E-05 |
| GO:0000156 | two-component response regulator activity | MF | 30 | 1.16E-13 |
| GO:0000155 | two-component sensor activity | MF | 26 | 2.96E-19 |
| GO:0008234 | cysteine-type peptidase activity | MF | 20 | 0.000358 |
| GO:0004673 | protein histidine kinase activity | MF | 20 | 2.13E-16 |
| GO:0016772 | transferase activity, transferring phosphorus-containing groups | MF | 17 | 6.48E-13 |
| GO:0004871 | signal transducer activity | MF | 10 | 0.000712 |
| GO:0004560 | alpha-L-fucosidase activity | MF | 7 | 1.34E-06 |
| GO:0016987 | sigma factor activity | MF | 6 | 0.000729 |
| GO:0008658 | penicillin binding | MF | 5 | 1.25E-05 |
| GO:0004482 | mRNA (guanine-N7-)-methyltransferase activity | MF | 4 | 0.000346 |
| GO:0016020 | membrane | CC | 134 | 2.05E-12 |
| GO:0019867 | outer membrane | CC | 4 | 0.00066 |
| GO:0019012 | virion | CC | 4 | 0.000346 |
| GO:0006810 | transport | BP | 101 | 7.72E-13 |
| GO:0006355 | regulation of transcription, DNA-dependent | BP | 67 | 1.65E-10 |
| GO:0000160 | two-component signal transduction system (phosphorelay) | BP | 36 | 1.83E-13 |
| GO:0018106 | peptidyl-histidine phosphorylation | BP | 20 | 2.13E-16 |
| GO:0016310 | phosphorylation | BP | 17 | 4.84E-14 |
| GO:0006633 | fatty acid biosynthetic process | BP | 6 | 0.000184 |
| GO:0042773 | ATP synthesis coupled electron transport | BP | 5 | 0.000227 |
| GO:0009273 | peptidoglycan-based cell wall biogenesis | BP | 5 | 3.76E-06 |
| GO:0006370 | mRNA capping | BP | 4 | 0.00066 |

Abbreviation: BP (Biological Process), CC (Cellular Component), MF (Molecular Function).
