# Supplementary material for: The invasive MED/Q Bemisia tabaci genome: a tale of gene loss and gene gain
Source: BMC Genomics. 2018 Jan 22;19:68. doi: 10.1186/s12864-018-4448-9 (PMC5778671; doi:10.1186/s12864-018-4448-9)
Supplement: Supplementary file 18 — Gene ontology of PSG in MED/Q and MEAM1/B (FDR < 0.05, P < 0.01). (DOCX 51 kb) [file 12864_2018_4448_MOESM18_ESM.docx]

**Table S6. Gene ontology of PSG in MED/Q and MEAM1/B (FDR < 0.05, P < 0.01)**

| # in MED/Q and MEAM1/B branch | | | | |
| --- | --- | --- | --- | --- |
| **GO ID** | **GO description** | **Type** | **Number of genes** | **P-value** |
| GO:0005216 | ion channel activity | MF | 3 | 0.001505 |
| GO:0004889 | acetylcholine-activated cation-selective channel activity | MF | 2 | 0.000357 |
| GO:0005230 | extracellular ligand-gated ion channel activity | MF | 2 | 0.006212 |
| GO:0008478 | pyridoxal kinase activity | MF | 1 | 0.004959 |
| GO:0003847 | 1-alkyl-2-acetylglycerophosphocholine esterase activity | MF | 1 | 0.004959 |
| GO:0045211 | postsynaptic membrane | CC | 2 | 0.000499 |
| GO:0007165 | signal transduction | BP | 6 | 0.000765 |
| GO:0006811 | ion transport | BP | 3 | 0.0054 |
| GO:0006164 | purine nucleotide biosynthetic process | BP | 2 | 0.002432 |
| GO:0009443 | pyridoxal 5'-phosphate salvage | BP | 1 | 0.004959 |
| GO:0019370 | leukotriene biosynthetic process | BP | 1 | 0.004959 |
| # in MED/Q branch | | | | |
| GO:0004672 | protein kinase activity | MF | 7 | 0.007833 |
| GO:0005198 | structural molecule activity | MF | 3 | 0.004526 |
| GO:0016849 | phosphorus-oxygen lyase activity | MF | 2 | 0.007953 |
| GO:0004357 | glutamate-cysteine ligase activity | MF | 1 | 0.009091 |
| GO:0004850 | uridine phosphorylase activity | MF | 1 | 0.009091 |
| GO:0004375 | glycine dehydrogenase (decarboxylating) activity | MF | 1 | 0.009091 |
| GO:0006468 | protein phosphorylation | BP | 8 | 0.002054 |
| GO:0035556 | intracellular signal transduction | BP | 4 | 0.006265 |
| GO:0009190 | cyclic nucleotide biosynthetic process | BP | 2 | 0.007953 |
| GO:0019370 | leukotriene biosynthetic process | BP | 1 | 0.009091 |
| GO:0006890 | retrograde vesicle-mediated transport, Golgi to ER | BP | 1 | 0.009091 |

Abbreviation: BP (Biological Process), CC (Cellular Component), MF (Molecular Function).
