# Supplementary material for: The invasive MED/Q Bemisia tabaci genome: a tale of gene loss and gene gain
Source: BMC Genomics. 2018 Jan 22;19:68. doi: 10.1186/s12864-018-4448-9 (PMC5778671; doi:10.1186/s12864-018-4448-9)
Supplement: Supplementary file 19 — Gene ontologies for gene families that have expanded number of members on MED/Q branch (FDR < 0.05, p < =0.000515776699029126). (DOCX 50 kb) [file 12864_2018_4448_MOESM19_ESM.docx]

**Table S7. Gene ontologies for gene families that have expanded number of members on MED/Q branch (FDR<0.05, p<=0.000515776699029126)**

| **GO ID** | **GO description** | **Type** | **Number of genes** | **P-value** |
| --- | --- | --- | --- | --- |
| GO:0022857 | transmembrane transporter activity | MF | 98 | 7.86E-101 |
| GO:0003824 | catalytic activity | MF | 82 | 0 |
| GO:0004970 | ionotropic glutamate receptor activity | MF | 59 | 2.95E-66 |
| GO:0005234 | extracellular-glutamate-gated ion channel activity | MF | 59 | 2.95E-66 |
| GO:0043169 | cation binding | MF | 57 | 2.14E-71 |
| GO:0003676 | nucleic acid binding | MF | 39 | 1.80E-05 |
| GO:0016491 | oxidoreductase activity | MF | 31 | 1.86E-05 |
| GO:0016747 | transferase activity, transferring acyl groups other than amino-acyl groups | MF | 21 | 2.14E-12 |
| GO:0004872 | receptor activity | MF | 20 | 1.27E-11 |
| GO:0005215 | transporter activity | MF | 20 | 0.000424 |
| GO:0016887 | ATPase activity | MF | 13 | 6.43E-05 |
| GO:0016021 | integral to membrane | CC | 100 | 0 |
| GO:0016020 | membrane | CC | 86 | 0 |
| GO:0055085 | transmembrane transport | BP | 99 | 1.17E-12 |
| GO:0005975 | carbohydrate metabolic process | BP | 57 | 0 |
| GO:0015074 | DNA integration | BP | 44 | 2.82E-13 |
| GO:0009058 | biosynthetic process | BP | 25 | 1.74E-11 |

Note: we calculated p-values by Fisher exact test for each GO category. We also corrected P-values by false discovery rate (FDR) considering the multiple testing on all the go terms. Abbreviation: BP (Biological Process), CC (Cellular Component), MF (Molecular Function).
