# Supplementary material for: The invasive MED/Q Bemisia tabaci genome: a tale of gene loss and gene gain
Source: BMC Genomics. 2018 Jan 22;19:68. doi: 10.1186/s12864-018-4448-9 (PMC5778671; doi:10.1186/s12864-018-4448-9)
Supplement: Supplementary file 21 — Gene ontologies for gene families that have expanded number of members on Bemisia tabaci branch (FDR < 0.05, p < =0.000879854368932039). (DOCX 53 kb) [file 12864_2018_4448_MOESM21_ESM.docx]

**Table S8. Gene ontologies for gene families that have expanded number of members on *Bemisia tabaci* branch (FDR<0.05, p<=0.000879854368932039)**

| **GO ID** | **GO description** | **Type** | **Number of genes** | **P-value** |
| --- | --- | --- | --- | --- |
| GO:0022857 | transmembrane transporter activity | MF | 142 | 2.04E-142 |
| GO:0003824 | catalytic activity | MF | 83 | 5.35E-12 |
| GO:0005506 | iron ion binding | MF | 79 | 2.97E-13 |
| GO:0020037 | heme binding | MF | 79 | 2.71E-13 |
| GO:0009055 | electron carrier activity | MF | 79 | 6.72E-13 |
| GO:0016705 | oxidoreductase activity, acting on paired donors, with incorporation or reduction of molecular oxygen | MF | 78 | 1.81E-55 |
| GO:0004970 | ionotropic glutamate receptor activity | MF | 59 | 3.74E-50 |
| GO:0005234 | extracellular-glutamate-gated ion channel activity | MF | 59 | 3.74E-50 |
| GO:0043169 | cation binding | MF | 57 | 1.06E-55 |
| GO:0016758 | transferase activity, transferring hexosyl groups | MF | 51 | 1.42E-38 |
| GO:0008234 | cysteine-type peptidase activity | MF | 50 | 1.98E-14 |
| GO:0005215 | transporter activity | MF | 36 | 2.79E-06 |
| GO:0016747 | transferase activity, transferring acyl groups other than amino-acyl groups | MF | 29 | 3.16E-13 |
| GO:0022891 | substrate-specific transmembrane transporter activity | MF | 24 | 2.51E-24 |
| GO:0042302 | structural constituent of cuticle | MF | 21 | 3.02E-08 |
| GO:0004872 | receptor activity | MF | 20 | 3.91E-07 |
| GO:0004497 | monooxygenase activity | MF | 19 | 4.42E-14 |
| GO:0003993 | acid phosphatase activity | MF | 15 | 2.20E-12 |
| GO:0004197 | cysteine-type endopeptidase activity | MF | 9 | 2.76E-06 |
| GO:0016021 | integral to membrane | CC | 144 | 3.78E-12 |
| GO:0016020 | membrane | CC | 104 | 1.47E-10 |
| GO:0055085 | transmembrane transport | BP | 143 | 2.59E-12 |
| GO:0055114 | oxidation-reduction process | BP | 83 | 5.24E-09 |
| GO:0005975 | carbohydrate metabolic process | BP | 57 | 6.57E-13 |
| GO:0008152 | metabolic process | BP | 53 | 8.37E-05 |
| GO:0006508 | proteolysis | BP | 51 | 1.92E-05 |
| GO:0015074 | DNA integration | BP | 45 | 1.02E-13 |
| GO:0009058 | biosynthetic process | BP | 25 | 2.88E-06 |
| GO:0050790 | regulation of catalytic activity | BP | 9 | 5.59E-08 |

Note: we calculated p-values by Fisher exact test for each GO category. We also corrected P-values by false discovery rate (FDR) considering the multiple testing on all the go terms. Abbreviation: BP (Biological Process), CC (Cellular Component), MF (Molecular Function).
