# Supplementary material for: The invasive MED/Q Bemisia tabaci genome: a tale of gene loss and gene gain
Source: BMC Genomics. 2018 Jan 22;19:68. doi: 10.1186/s12864-018-4448-9 (PMC5778671; doi:10.1186/s12864-018-4448-9)
Supplement: Supplementary file 22 — Gene ontology over-representation of gene families contracted on Bemisia tabaci branch (FDR < 0.05, p < =0.000572390572). (DOCX 49 kb) [file 12864_2018_4448_MOESM22_ESM.docx]

**Table S9. Gene ontology over-representation of gene families contracted on *Bemisia tabaci* branch (FDR<0.05, p<=0.000572390572)**

| **GO ID** | **GO description** | **Type** | **Number of genes** | **P-value** |
| --- | --- | --- | --- | --- |
| GO:0003723 | RNA binding | MF | 10 | 9.14E-14 |
| GO:0003964 | RNA-directed DNA polymerase activity | MF | 10 | 5.75E-17 |
| GO:0004252 | serine-type endopeptidase activity | MF | 3 | 0.000111 |
| GO:0006278 | RNA-dependent DNA replication | BP | 10 | 5.75E-17 |

Abbreviation: BP (Biological Process), CC (Cellular Component), MF (Molecular Function).
