# Supplementary material for: The invasive MED/Q Bemisia tabaci genome: a tale of gene loss and gene gain
Source: BMC Genomics. 2018 Jan 22;19:68. doi: 10.1186/s12864-018-4448-9 (PMC5778671; doi:10.1186/s12864-018-4448-9)
Supplement: Supplementary file 23 — Gene ontology over-representation of gene families contracted on Bemisia tabaci branch (FDR < 0.05, p < =0.000572390572). (DOCX 49 kb) [file 12864_2018_4448_MOESM23_ESM.docx]

**Table S10. Gene ontology over-representation of gene families contracted on *Bemisia tabaci* branch (FDR<0.05, p<=0.000572390572)**

| **GO ID** | **GO description** | **Type** | **Number of genes** | **P-value** |
| --- | --- | --- | --- | --- |
| GO:0003723 | RNA binding | MF | 5 | 3.12E-05 |
| GO:0003964 | RNA-directed DNA polymerase activity | MF | 5 | 8.97E-07 |
| GO:0015074 | DNA integration | BP | 11 | 2.11E-18 |
| GO:0006278 | RNA-dependent DNA replication | BP | 5 | 8.97E-07 |

Abbreviation: BP (Biological Process), CC (Cellular Component), MF (Molecular Function).
