# Supplementary material for: The invasive MED/Q Bemisia tabaci genome: a tale of gene loss and gene gain
Source: BMC Genomics. 2018 Jan 22;19:68. doi: 10.1186/s12864-018-4448-9 (PMC5778671; doi:10.1186/s12864-018-4448-9)
Supplement: Supplementary file 24 — Immune system-related and virus transport genes in phloem- and blood-feeding insects. (DOCX 56 kb) [file 12864_2018_4448_MOESM24_ESM.docx]

**Table S11. Immune system-related and virus transport genes in phloem- and blood-feeding insects**

| **Function classification** | **Gene** | **plant phloem sap specialist** | | | **animal blood specialist** | | |
| --- | --- | --- | --- | --- | --- | --- | --- |
|  |  | ***B.tabaci**** | ***A.pisum*** | ***N.lugens*** | ***A.gambiae*** | ***R. prolixus*** | ***P. humanus*** |
| **Pattern recognition molecules** | PGRP | 1 | 0 | 2 | 7 | 3 | 0 |
|  | hemocytin | 3 | 1 | 1 | 0 | 2 | 3 |
|  | GNBP | 4 | 2 | 7 | 7 | 2 | 0 |
|  | galectin | 5 | 2 | 2 | 10 | 3 | 0 |
|  | ester | 1 | 0 | 1 | 1 | 0 | 0 |
|  | dscam | 11 | 1 | 9 | 1 | 1 | 1 |
|  | draper | 1 | 1 | 1 | 1 | 1 | 1 |
|  | c-type lectin | 12 | 10 | 9 | 25 | 5 | 15 |
| **Toll pathway** | Toll | 7 | 5 | 6 | 10 | 4 | 8 |
|  | cactus | 1 | 1 | 1 | 1 | 1 | 1 |
|  | Myd88 | 1 | 1 | 2 | 1 | 0 | 1 |
|  | Spatzle | 8 | 4 | 8 | 6 | 1 | 0 |
|  | Pelle | 2 | 1 | 1 | 1 | 1 | 1 |
|  | Tube | 1 | 1 | 1 | 1 | 1 | 1 |
|  | Drosal/Dif | 1 | 1 | 1 | 1 | 2 | 2 |
|  | Tollip | 1 | 1 | 1 | 2 | 1 | 0 |
| **IMD pathway** | Dredd | 0 | 0 | 1 | 1 | 0 | 0 |
|  | IMD | 0 | 0 | 1 | 1 | 0 | 0 |
|  | Relish | 0 | 0 | 1 | 1 | 0 | 0 |
|  | Tak1 | 0 | 0 | 1 | 1 | 0 | 0 |
|  | IAP2 | 0 | 1 | 1 | 1 | 1 | 1 |
|  | Ubc13 | 0 | 1 | 1 | 1 | 1 | 1 |
|  | TRAF | 0 | 2 | 2 | 1 | 1 | 1 |
|  | Ikk | 0 | 1 | 2 | 2 | 0 | 1 |
|  | Fadd | 0 | 0 | 0 | 1 | 0 | 0 |
|  | Tab2 | 1 | 1 | 1 | 1 | 0 | 1 |
| **JAK-STAT pathway** | JAK | 1 | 1 | 1 | 1 | 1 | 1 |
|  | Pias | 1 | 2 | 1 | 1 | 0 | 0 |
|  | socs | 2 | 5 | 5 | 1 | 1 | 2 |
|  | STAT | 1 | 2 | 1 | 2 | 1 | 1 |
|  | Dome | 1 | 1 | 1 | 1 | 1 | 1 |
| **proPO cascade** | lysozyme | 3 | 3 | 8 | 8 | 4 | 3 |
|  | Serpin protease inhibitor | 8 | 16 | 9 | 17 | 7 | 11 |
|  | Clip-domain protease | 4 | 6 | 12 | 58 | 4 | 5 |
| **Immune effector** | Attacin | 0 | 0 | 0 | 1 | 0 | 0 |
|  | Cecropin | 0 | 0 | 0 | 4 | 0 | 0 |
|  | Defensin | 4 | 0 | 2 | 4 | 2 | 2 |
|  | Dipericin | 0 | 0 | 0 | 0 | 0 | 0 |
|  | thaumatin | 1 | 8 | 2 | 0 | 0 | 0 |
|  | drosocin | 0 | 0 | 0 | 0 | 0 | 0 |
|  | Drosomycin | 0 | 0 | 0 | 0 | 0 | 0 |
|  | knottin | 3 | 0 | 0 | 0 | 0 | 0 |
|  | NOS | 1 | 1 | 1 | 1 | 3 | 2 |
| **Autophagy** | PI3K | 6 | 1 | 2 | 3 | 2 | 2 |
|  | Rhed | 2 | 2 | 2 | 2 | 2 | 2 |
|  | TSC | 2 | 1 | 2 | 1 | 1 | 1 |
|  | Akt | 1 | 1 | 1 | 1 | 1 | 1 |
|  | TOR | 2 | 2 | 2 | 3 | 2 | 2 |
|  | Atg1 | 1 | 1 | 1 | 1 | 1 | 1 |
|  | Atg2 | 1 | 1 | 1 | 1 | 1 | 1 |
|  | Atg3 | 1 | 1 | 1 | 1 | 1 | 1 |
|  | Atg4 | 1 | 1 | 0 | 2 | 1 | 1 |
|  | Atg5 | 0 | 1 | 1 | 1 | 1 | 1 |
|  | Atg6 | 1 | 1 | 2 | 2 | 1 | 1 |
|  | Atg7 | 1 | 3 | 2 | 3 | 4 | 1 |
|  | Atg8 | 1 | 2 | 1 | 1 | 1 | 2 |
|  | Atg9 | 3 | 2 | 1 | 2 | 1 | 1 |
|  | Atg10 | 1 | 3 | 2 | 5 | 1 | 1 |
|  | Atg11 | 0 | 0 | 0 | 0 | 0 | 0 |
|  | Atg12 | 1 | 1 | 0 | 2 | 1 | 1 |
|  | Atg13 | 1 | 1 | 0 | 2 | 1 | 1 |
|  | Atg14 | 1 | 1 | 1 | 1 | 1 | 1 |
|  | Atg15 | 0 | 0 | 0 | 0 | 0 | 0 |
|  | Atg16 | 1 | 1 | 1 | 1 | 1 | 1 |
|  | Atg17 | 0 | 1 | 1 | 1 | 1 | 1 |
|  | Atg18 | 0 | 3 | 4 | 4 | 3 | 3 |
|  | Atg101 | 1 | 1 | 2 | 0 | 1 | 1 |
| **pi- si- mi-RNA pathways** | Dicer1 | 1 | 2 | 1 | 1 | 1 | 1 |
|  | Dicer2 | 2 | 1 | 1 | 1 | 1 | 1 |
|  | Drosha | 1 | 1 | 1 | 1 | 1 | 1 |
|  | Ago1 | 1 | 1 | 1 | 1 | 0 | 1 |
|  | Ago2 | 2 | 2 | 1 | 1 | 1 | 1 |
|  | Ago3 | 1 | 1 | 1 | 1 | 1 | 1 |
|  | piwi | 5 | 5 | 2 | 2 | 3 | 1 |
|  | Armi | 1 | 1 | 1 | 1 | 1 | 1 |
|  | Pasha | 1 | 1 | 1 | 1 | 1 | 1 |
|  | Vig | 1 | 0 | 1 | 1 | 0 | 1 |
|  | Exportin5 | 1 | 1 | 1 | 1 | 1 | 1 |
|  | Shu | 1 | 1 | 1 | 1 | 1 | 1 |
|  | Qin | 1 | 1 | 1 | 1 | 1 | 1 |
|  | TSN | 1 | 2 | 1 | 2 | 1 | 1 |
|  | Ars2 | 1 | 1 | 1 | 1 | 1 | 1 |
|  | CBC | 1 | 1 | 3 | 1 | 1 | 1 |
|  | Belle/Cap | 1 | 1 | 2 | 1 | 1 | 2 |
|  | Blanks | 2 | 0 | 1 | 0 | 0 | 0 |
|  | Trsn | 0 | 0 | 1 | 1 | 0 | 1 |
|  | Tis11 | 1 | 1 | 1 | 1 | 0 | 0 |
|  | R2D2 | 5 | 5 | 2 | 1 | 3 | 2 |
|  | Yb | 1 | 1 | 1 | 1 | 1 | 1 |
|  | Nbr | 1 | 1 | 1 | 1 | 1 | 1 |
|  | GW | 1 | 0 | 1 | 3 | 0 | 1 |
|  | Me31B | 1 | 1 | 1 | 1 | 1 | 1 |
|  | HPS4 | 1 | 1 | 1 | 1 | 1 | 1 |
|  | Ge-1/EDC4 | 1 | 1 | 0 | 1 | 1 | 1 |
|  | Zuc | 2 | 1 | 0 | 1 | 3 | 1 |
| **Apoptosis** | Reaper | 0 | 0 | 0 | 0 | 0 | 0 |
|  | IAP1 | 2 | 3 | 3 | 4 | 2 | 2 |
|  | Deterin/IAP3 | 1 | 2 | 2 | 1 | 1 | 2 |
|  | Ark | 0 | 1 | 0 | 1 | 1 | 0 |
|  | caspase-1 | 2 | 3 | 2 | 4 | 1 | 2 |
|  | Dronc | 1 | 2 | 0 | 1 | 1 | 1 |
| **Virus transport** | actin | 15 | 16 | 13 | 14 | 3 | 12 |
|  | GAPDH | 4 | 1 | 1 | 1 | 4 | 1 |
|  | cyclophilin | 20 | 20 | 15 | 11 | 11 | 13 |

“*”: *B.tabaci*: *Bemisia tabaci*, *N. lugens*: *Nilaparvata lugens*, *A. pisum*: *Acyrthosiphum pisum*, *R prolixus*: *Rhodnius prolixus*, *P. humanus*: *Pediculus humanus*, *A. gambiae*: *Anopheles gambiae*
