# Supplementary material for: The invasive MED/Q Bemisia tabaci genome: a tale of gene loss and gene gain
Source: BMC Genomics. 2018 Jan 22;19:68. doi: 10.1186/s12864-018-4448-9 (PMC5778671; doi:10.1186/s12864-018-4448-9)
Supplement: Supplementary file 26 — Genes involved in B vitamin biosynthesis in MED/Q. (DOCX 50 kb) [file 12864_2018_4448_MOESM26_ESM.docx]

**Table S12. Genes involved in B vitamin biosynthesis in MED/Q**

| **Pathway** | **Gene id** | **KO Name** | **EC number** |
| --- | --- | --- | --- |
| NAD | BTA020960.1 | E6.3.5.1, NADSYN1, QNS1, nadE | 6.3.5.1 |
| Biotin | BTA024803.1 | E2.6.1.62, bioA | 2.6.1.62 |
|  | BTA023651.1 | E2.6.1.62, bioA | 2.6.1.62 |
|  | BTA004814.1 | E2.6.1.62, bioA | 2.6.1.62 |
|  | BTA022657.1 | E2.6.1.62, bioA | 2.6.1.62 |
| CoA | BTA010941.1 | E2.6.1.16, glmS | 2.6.1.16 |
|  | BTA007140.1 | E2.6.1.16, glmS | 2.6.1.16 |
|  | BTA000221.1 | coaW | 2.7.1.33 |
|  | BTA029734.1 | coaW | 2.7.1.33 |
|  | BTA011878.1 | coaW | 2.7.1.33 |
|  | BTA020903.1 | coaW | 2.7.1.33 |
|  | BTA002606.1 | PPCS, coaB | 6.3.2.5 |
|  | BTA020890.1 | PPCDC, coaC | 4.1.1.36 |
| Folate | BTA019073.1 | E3.5.4.16, folE | 3.5.4.16 |
|  | BTA022091.1 | E3.5.4.16, folE | 3.5.4.16 |
|  | BTA026052.1 | E3.5.4.16, folE | 3.5.4.16 |
|  | BTA010678.1 | folA | 1.5.1.3 |
| CoA | BTA006612.1 | E3.1.3.1, phoA, phoB | 3.1.3.1 |
|  | BTA008972.1 | phoD | 3.1.3.1 |
|  | BTA017150.1 | E3.1.3.1, phoA, phoB | 3.1.3.1 |
|  | BTA023249.1 | E3.1.3.1, phoA, phoB | 3.1.3.1 |
|  | BTA027509.1 | E3.1.3.1, phoA, phoB | 3.1.3.1 |
|  | BTA001657.1 | E3.1.3.1, phoA, phoB | 3.1.3.1 |
| Heme | BTA006330.1 | EARS, gltX | 6.1.1.17 |
|  | BTA004894.1 | hemB, ALAD | 4.2.1.24 |
|  | BTA017745.1 | hemB, ALAD | 4.2.1.24 |
|  | BTA026808.1 | hemB, ALAD | 4.2.1.24 |
|  | BTA000296.1 | hemC, HMBS | 2.5.1.61 |
|  | BTA023640.2 | hemC, HMBS | 2.5.1.61 |
|  | BTA029306.1 | hemC, HMBS | 2.5.1.61 |
|  | BTA007218.1 | hemD, UROS | 4.2.1.75 |
|  | BTA020659.1 | hemD, UROS | 4.2.1.75 |
|  | BTA016634.1 | hemE, UROD | 4.1.1.37 |
|  | BTA018685.1 | hemE, UROD | 4.1.1.37 |
|  | BTA027323.1 | hemF, CPOX | 1.3.3.3 |
|  | BTA028283.1 | hemF, CPOX | 1.3.3.3 |
|  | BTA009177.1 | hemH, FECH | 4.99.1.1 |
| VB6 | BTA012643.1 | serC | 2.6.1.52 |
|  | BTA012645.1 | serC | 2.6.1.52 |
|  | BTA011556.1 | pdxH, PNPO | 1.4.3.5 |
