# Supplementary material for: The invasive MED/Q Bemisia tabaci genome: a tale of gene loss and gene gain
Source: BMC Genomics. 2018 Jan 22;19:68. doi: 10.1186/s12864-018-4448-9 (PMC5778671; doi:10.1186/s12864-018-4448-9)
Supplement: Supplementary file 27 — Genes involved in B vitamin biosynthesis in Candidatus Hamiltonella defense. (DOCX 51 kb) [file 12864_2018_4448_MOESM27_ESM.docx]

**Table S13. Genes involved in B vitamin biosynthesis in *Candidatus* Hamiltonella defense**

| **Pathway** | **Gene id** | **KO Name** | **EC number** |
| --- | --- | --- | --- |
| NAD | H44GL000523 | nadB | 1.4.3.16 |
|  | H44GL001328 | nadA | 2.5.1.72 |
|  | H44GL001267 | E2.4.2.19, nadC | 2.4.2.19 |
|  | H44GL001347 | nadD | 2.7.7.18 |
|  | H44GL000355 | E6.3.5.1, NADSYN1, QNS1, nadE | 6.3.5.1 |
| Biotin | H44GL000999 | bioF | 2.3.1.47 |
|  | H44GL000873 | E2.6.1.62, bioA | 2.6.1.62 |
|  | H44GL001001 | bioD | 6.3.3.3 |
|  | H44GL000998 | E2.8.1.6, bioB | 2.8.1.6 |
| Ribofalvin | H44GL000255 | ribA, RIB1 | 3.5.4.25 |
|  | H44GL000174 | ribD | 3.5.4.26,1.1.1.193 |
|  | H44GL001016 | sixA | 3.1.3.- |
|  | H44GL000267 | ribB, RIB3 | 4.1.99.12 |
|  | H44GL000624 | ribH, RIB4 | 2.5.1.78 |
|  | H44GL000985 | ribE, RIB5 | 2.5.1.9 |
|  | H44GL000594 | ribF | 2.7.1.26,2.7.7.2 |
| CoA | H44GL000336 | E2.7.7.22, manC | 2.7.7.22 |
|  | H44GL000365 | E2.6.1.16, glmS | 2.6.1.16 |
|  | H44GL001319 | coaA | 2.7.1.33 |
|  | H44GL000120 | coaBC, dfp | 4.1.1.36，6.3.2.5 |
|  | H44GL000737 | E2.7.7.3A, coaD, kdtB | 2.7.7.3 |
|  | H44GL000890 | coaE | 2.7.1.24 |
| Folate | H44GL000390 | E3.5.4.16, folE | 3.5.4.16 |
|  | H44GL000468 | folB | 4.1.2.25 |
|  | H44GL001383 | folK | 2.7.6.3 |
|  | H44GL001640 | folP | 2.5.1.15 |
|  | H44GL001210 | folC | 6.3.2.12，6.3.2.17 |
|  | H44GL001390 | folA | 1.5.1.3 |
| Heme | H44GL000424 | EARS, gltX | 6.1.1.17 |
|  | H44GL001559 | hemA | 1.2.1.70 |
|  | H44GL000911 | hemC, HMBS | 2.5.1.61 |
|  | H44GL000910 | hemD, UROS | 4.2.1.75 |
|  | H44GL000847 | hemE, UROD | 4.1.1.37 |
|  | H44GL001250 | hemF, CPOX | 1.3.3.3 |
|  | H44GL000788 | hemG | 1.3.3.4 |
|  | H44GL000349 | hemH, FECH | 4.99.1.1 |
|  | Hamiltonella.defense GL001745* | hemL | 5.4.3.8 |
|  | Hamiltonella.defense GL001746* | hemL | 5.4.3.8 |
|  | Hamiltonella.defense GL000574* | hemB, ALAD | 4.2.1.24 |
| Thiamine | H44GL001150 | pyrH | 2.7.4.22 |
|  | H44GL001186 | uppS | 2.5.1.31 |
|  | H44GL000626 | thiL | 2.7.4.16 |
| VB6 | H44GL001215 | pdxB | 1.1.1.290 |
|  | H44GL000883 | serC, PSAT1 | 2.6.1.52 |
|  | H44GL001219 | pdxA | 1.1.1.262 |
|  | H44GL000102 | pdxJ | 2.6.99.2 |
|  | H44GL000942 | pdxH, PNPO | 1.4.3.5 |

*: from Rao, Q., et al. (2012). "Draft genome sequence of “Candidatus Hamiltonella defensa,” an endosymbiont of the whitefly Bemisia tabaci." Journal of bacteriology 194(13): 3558-3558.
