# Supplementary material for: The invasive MED/Q Bemisia tabaci genome: a tale of gene loss and gene gain
Source: BMC Genomics. 2018 Jan 22;19:68. doi: 10.1186/s12864-018-4448-9 (PMC5778671; doi:10.1186/s12864-018-4448-9)
Supplement: Supplementary file 29 — Horizontally transferred genes involved in amino acid biosynthesis in MED/Q. (DOCX 50 kb) [file 12864_2018_4448_MOESM29_ESM.docx]

**Table S14. Horizontally transferred genes involved in amino acid biosynthesis in MED/Q**

| **Gene ID** | **Name** | **EC number** | **Co^a^** | **In^b^** | **In^c^** | **Pseudogenes** | **Origin** |
| --- | --- | --- | --- | --- | --- | --- | --- |
| BTA005852.1 | argH | 4.3.2.1 | Yes |  |  | Yes | *Plautia stali symbiont* (Bacteria) |
| BTA005853.1 | argH | 4.3.2.1 | Yes |  |  |  | *Plautia stali symbiont* (Bacteria) |
| BTA005854.1 | argG | 6.3.4.5 | Yes |  |  | Yes | *Pantoea sp.* (Bacteria) |
| BTA007760.1 | dapF | 5.1.1.7 | Yes | Yes |  |  | *Pantoea ananatis* (Bacteria) |
| BTA013028.1 | dapF | 5.1.1.7 | Yes | Yes | Yes |  | *Pantoea ananatis* (Bacteria) |
| BTA026180.1 | lysA | 4.1.1.20 | Yes |  |  |  | *Isosphaera pallida* (Bacteria) |
| BTA029276.1 | dapB | 1.17.1.8 | Yes |  |  |  | *Rickettsia bellii* (Bacteria) |
| BTA023603.1 | E3.1.3.15B (HisB) | 3.1.3.15 |  | Yes |  |  | *Pseudomonas syringae* (Bacteria) |
| BTA020874.1 | E5.4.99.5 | 5.4.99.5 | Yes | Yes | Yes | Yes | *Enterobacter mori* (Bacteria) |
| BTA007752 | SCLY | 4.4.1.16 | Yes | Yes | Yes |  | *Eutypa lata* (fungi) |
| BTA014319.1 | E4.3.1.19 | 4.3.1.19 | Yes | Yes | Yes |  | *Polysphondylium pallidum PN500* (fungi) |

“a”: Co-assemble with insect gene

“b”: Intron

“c’”: 5' UTR intron
