# Supplementary material for: The invasive MED/Q Bemisia tabaci genome: a tale of gene loss and gene gain
Source: BMC Genomics. 2018 Jan 22;19:68. doi: 10.1186/s12864-018-4448-9 (PMC5778671; doi:10.1186/s12864-018-4448-9)
Supplement: Supplementary file 30 — Comparison of transaminases in three symbiotic systems Bemisia tabaci/Portiera, Acyrthosiphum pisum/Buchnera and Nilaparvata lugens/Yeast-like. (DOCX 50 kb) [file 12864_2018_4448_MOESM30_ESM.docx]

**Table S15. Comparasion of transaminase number in three symbiotic systems *Bemisia tabaci*/Portiera, *Acyrthosiphum pisum*/Buchnera and *Nilaparvata lugens*/Yeast-like**

|  | **EC number** | ***B.tabaci-Portiera*** | | ***Aphid-Buchnera*** | | ***N.lugens-Yeast-like*** | |
| --- | --- | --- | --- | --- | --- | --- | --- |
| **Gene name** |  | ***B.tabaci*** | ***Portiera*** | ***A.pisum*** | ***Buchnera*** | ***N.lugens*** | **yeast-like** |
| aspartate transaminase | 2.6.1.1 | 6 | 0 | 4 | 0 | 3 | 2 |
| alanine transaminase | 2.6.1.2 | 1 | 0 | 1 | 0 | 4 | 1 |
| tyrosine transaminase | 2.6.1.5 | 2 | 0 | 0 | 0 | 2 | 0 |
| kynurenine-oxoglutarate transaminase | 2.6.1.7 | 0 | 0 | 2 | 0 | 0 | 3 |
| histidinol-phosphate transaminase | 2.6.1.9 | 0 | 1 | 0 | 1 | 0 | 1 |
| acetylornithine transaminase | 2.6.1.11 | 0 | 1 | 0 | 1 | 0 | 1 |
| ornithine aminotransferase | 2.6.1.13 | 4 | 0 | 1 | 0 | 3 | 1 |
| glutamine-fructose-6-phosphate transaminase | 2.6.1.16 | 1 | 0 | 1 | 1 | 1 | 0 |
| succinyldiaminopimelate transaminase | 2.6.1.17 | 1 | 0 | 0 | 1 | 0 | 0 |
| 4-aminobutyrate transaminase | 2.6.1.19 | 0 | 0 | 1 | 0 | 0 | 1 |
| branched-chain-amino-acid transaminase | 2.6.1.42 | 1 | 0 | 1 | 0 | 3 | 3 |
| alanine-glyoxylate transaminase | 2.6.1.44 | 0 | 0 | 3 | 0 | 0 | 1 |
| phosphoserine transaminase | 2.6.1.52 | 3 | 0 | 1 | 1 | 1 | 1 |
| undefined | 2.6.1.- | 3 | 0 | 1 | 0 | 1 | 0 |
| total |  | 22 | 2 | 16 | 5 | 18 | 15 |
