# Supplementary material for: The invasive MED/Q Bemisia tabaci genome: a tale of gene loss and gene gain
Source: BMC Genomics. 2018 Jan 22;19:68. doi: 10.1186/s12864-018-4448-9 (PMC5778671; doi:10.1186/s12864-018-4448-9)
Supplement: Supplementary file 32 — Primers used in this study. (DOCX 20 kb) [file 12864_2018_4448_MOESM32_ESM.docx]

**Table S16.** Primers used in this study.

| **Putative**  **Gene** | **Primers**  **5′-3′^a^** | **Annealing temperature (°C)** | **Product (bp)** | **Amplification efficiency (%)** |
| --- | --- | --- | --- | --- |
| BTCYP304G2  Full length | F- ATGCATTGTTATTTTAAAATTTCGAGC  R- AGTGCCCCGAGGAACAATTTTC | 64  64 | 1521 | - |
| BTCYP304G2  qPCR | F- TTAGTCGCCAAACTTCGCTCC  R- CACTGCGCCGTCCATAACC | 62  62 | 133 | 101 |
| BTCYP304G2  RNAi | F- AGCATCTGCGAGGCTACATT  R- AACGACAATTGGCTTCAACC | 57  57 | 573 | - |
| BTCYP402C9  Full length | F- ATGCATCTTTTGGAGACCCTGTTT  R- GTATGCGCGAACCTCTCGAGG | 63  64 | 1566 | - |
| BTCYP402C9  qPCR | F- GAGGCTAAGCGGTGAGGGATT  R- CTGTACAAAGTTCATTGCCACCTC | 62  61 | 127 | 95 |
| BTCYP402C9  RNAi | F- GAAAGTTTTGCTGAGCCAGG  R- AAGGGTCGACAACAATACGC | 57  57 | 592 | - |
| BTCYP4CR2  Full length | F- ATGTATGAGTATTTCGAACCCTGGC  R- ATTCCTTTTTTCGAGTCGAATATGAA | 63  61 | 1212 | - |
| BTCYP4CR2  qPCR | F- CGTTGCGACACTACCCGTCTG  R- GGCAGGGATGACATAACCACC | 63  61 | 89 | 98 |
| BTCYP4CR2  RNAi | F- TGAGTATTTCGAACCCTGGC  R- ATCCCCTCTTCAAAATGGCT | 57  57 | 332 | - |
| BTCYP4G69  Full length | F- ATGGCTAAAACAAGCCTCAGCTC  R- ATTCTTTGGTTTTGGTGTCATTTTC | 62  61 | 1710 | - |
| BTCYP4G69  qPCR | F- ATGGAAAGGTGCATAATGGAGAC  R- AACGGGACGAAAGCATAGTAGTG | 60  60 | 245 | 98 |
| BTCYP4G69  RNAi | F- GCTAGCAGCCCAATTTCAAG  R- CTCAGAGCTCGCCATTTTTC | 57  57 | 306 | - |
| BTCYP6CX4  Full length | F- ATGGGGAACCTGGTGGAGTTG  R- AATTCTCTTTTTGAGTCGAAGCCAT | 63  62 | 1596 | - |
| BTCYP6CX4  qPCR | F- GTGACCCGACTTTCTTCCCTG  R- CGTTGAGCGATGCAGAATCTAG | 60  60 | 130 | 100 |
| BTCYP6CX4  RNAi | F- CTTACCTTTCTCGCTGGACG  R- AGCCAACTGCGAACATCTCT | 57  57 | 520 | - |
| BTCYP6DB3  Full length | F- ATGTTCGAGTTCCTGCAAAAAGT  R- TAATGGTTTAATATTCAACCAAATCCC | 60  62 | 1536 | - |
| BTCYP6DB3  qPCR | F- CCAGCAGCCTTCTCACCTTC  R- GCTTGCGAAGTGTCTCGTTGA | 60  61 | 174 | 96 |
| BTCYP6DB3  RNAi | F- TCAACCACGTTTGTATCCGA  R- CATCTTGATTGGCGAAGGAT | 57  57 | 468 | - |
| BTCYP6DV6  Full length | F- ATGATTCTCGAGCTTCTCCTTCC  R- AGCAGCAGGCCTCCTTTTCAAC | 61  64 | 1527 | - |
| BTCYP6DV6  qPCR | F- ACTGGAGGAACTACACGCACTG  R- TCGTCGTAGAACCGCTTGATAG | 60  62 | 115 | 104 |
| BTCYP6DV6  RNAi | F- CTCAAAACAAAGCTGAGCCC  R- CACCTGGTCCCCCTTTTAAT | 57  57 | 352 | - |
| BTCYP6DW2 Full length | F- ATGCTGTCAGCGGTGACCGT  R- CTTTGCGCGTTTTTGGAATTTTAG | 63  64 | 1512 | - |
| BTCYP6DW2  qPCR | F- CAGGTCCTCCGCTCCATCC  R- CGCTTGTCCTGGTTCTGCTC | 62  60 | 134 | 103 |
| BTCYP6DW2  RNAi | F- AAACGACGAAGAACAAGGC  R- GAACTTGAGCATCAGCTCCC | 57  57 | 392 | - |
| BTCYP6EM1  Full length | F- ATGGGTCCTTTTACGATAAGTTTGAT  R- GATATCATGACGAAGAGATTGATACCAC | 61  62 | 1533 | - |
| BTCYP6EM1  qPCR | F- GCAAACGCTCTGCTCGGAAAC  R- GCTTAAACTCGCTGTTCTCACCCT | 64  63 | 140 | 100 |
| BTCYP6EM1  RNAi | F- TGTTGATGAATCTTCGCAGC  R- CTCTCATCCCCTTTTCCACA | 57  57 | 396 | - |
| BTGSTM1  Full length | F- ATGGCTCTGCTGTCGCAGATC  R- CTACAAGACATATAGGATAGTCTGTA  CAAGC | 62  60 | 459 | - |
| BTGSTM1  qPCR | F- GGAGCCAAAGAATCAAGAAAGG  R- ATCGTTCTGGTGAGCCCTTCTA | 60  61 | 122 | 97 |
| BTGSTM1  RNAi | F- CCGTCTTCACGGCGTATATT  R- GTGCGGGTTGAGGTATGACT | 57  57 | 348 | - |
| BTGSTD6  Full length | F- ATGTTCAAATTCACACAACGTTTGG  R- CGAGTTAGTTTTCACTTTCGTCTTTTGC | 63  61 | 747 | - |
| BTGSTD6  qPCR | F- ATGCCGTTTAGCCCTCCTTGT  R- GAATCCGTCGTCATCTATAACAGG | 62  60 | 165 | 98 |
| BTGSTD6  RNAi | F- AGATGGGGCGTATTGATCTG  R- AAATCCAGTGCTTGGTCCAC | 57  57 | 307 | - |
| BTGSTD9  Full length | F- ATGACCGTAGACATTTATCACATCG  R- TTAAAGTGACCTAAGATGTTCCCAG | 60  59 | 651 | - |
| BTGSTD9  qPCR | F- GCCTCCATCGTTACCATCATCT  R- CACTTGTCGTACCAACGGCTG | 61  61 | 89 | 101 |
| BTGSTD9  RNAi | F- CATCGGACCTAGTCCACCAT  R- CCAACGGCTGATGTTAGGAT | 57  57 | 532 | - |

^a^F, forward primer; R, reverse primer
